# Discrimination for geographical origin of *Panax quinquefolius* L. using UPLC Q‐Orbitrap MS‐based metabolomics approach

**DOI:** 10.1002/fsn3.3461

**Published:** 2023-07-10

**Authors:** Shifeng Pang, Xiangmin Piao, Xiaohao Zhang, Xiaolin Chen, Hao Zhang, Yinping Jin, Zheng Li, Yingping Wang

**Affiliations:** ^1^ Institute of Special Animal and Plant Sciences Chinese Academy of Agricultural Sciences Changchun China; ^2^ Jilin Provincial Key Laboratory of Traditional Chinese Medicinal Materials Cultivation and Propagation Changchun China; ^3^ State‐Local Joint Engineering Research Center of Ginseng Breeding and Application Jilin Agricultural University Changchun China; ^4^ Department of Cardiology The Second Hospital of Jilin University Changchun China; ^5^ Ginseng Antler Office of Jilin Province (TCM Development Center of Department of Agriculture and Rural Affairs of Jilin Province) Changchun China

**Keywords:** geographical origin discrimination, metabolomics, *Panax quinquefolius* L., UPLC Q–Orbitrap MS

## Abstract

American ginseng, *Panax quinquefolius* L., is an important medicinal plant with multiple pharmacological effects and high nutritional value. American ginseng from different geographical origins varies in quality and price. However, there was no approach for discriminating American ginseng from different geographical origins to date. In this study, a metabolomic method based on the UPLC–Orbitrap fusion platform was established to comprehensively determine and analyze metabolites of American ginseng from America and Canada, Heilongjiang, Jilin, Liaoning, and Shandong provinces in China. A total of 382 metabolites were detected, including 230 saponins, 30 amino acids and derivatives, 27 organic acids and derivatives, 25 lipids, 17 carbohydrates and derivatives, 10 phenols, 8 nucleotides, and derivatives, as well as 35 other metabolites. Metabolite differences between North America and Asia producing areas were more obvious than within Asia. Twenty metabolites, contributed most to the differentiation of producing areas, were identified as potential markers with prediction accuracy higher than 91%. The results provide new insights into the metabolite composition of American ginseng from different origins, which will help discriminate origins and promote quality control of American ginseng.

## Graphical abstract

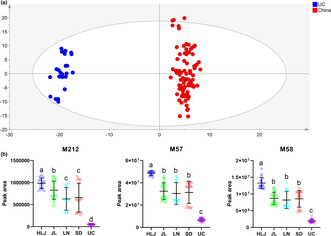

## 1 INTRODUCTION

American ginseng (*Panax quinquefolius* L.), native to the eastern temperate forest areas of North America, was first discovered in Quebec, Canada. American ginseng began to be cultivated in China since the 1980s (McGraw et al., 2013). With over 40 years of development, China had become the third largest country for American ginseng cultivation and the current major producing areas are Heilongjiang, Jilin, Liaoning, and Shandong Provinces (Huang et al., 2013).

As a well‐known medicinal plant, American ginseng is consumed as dietary supplements and functional food with a large market demand. It is famous for a wide range of pharmacological effects, such as anticancer, antioxidative, antiaging, antifatigue, and enhancement of memory and immunity effects (Cheong et al., 2014; Hwang et al., 2014; Kim et al., 2018; Kwok, 2011; Qi et al., 2010, 2011; Riaz et al., 2019; Tan et al., 2013). Various chemical constituents, including ginsenosides, lipids, polysaccharides, organic acids, amino acids, phenolic acids, and vitamins, have been identified in American ginseng, which was responsible for multiple efficacy (Guo et al., 2015; Hou, 1977; Lin et al., 2019; Wang et al., 2015).

Medicinal plants of different origins varied significantly in bioactive ingredient, directly affecting their quality (Liu et al., 2017). For example, *Carthamus tinctorius* L. from Hunan province had higher contents of hydroxysafflor yellow A and succinate and showed stronger antioxidant, anticoagulant, and cardiovascular protection effects than other provinces (Lu et al., 2019). Higher amounts of ophiopogonones were observed in the extract of Ophiopogon japonicus from Zhejiang, which showed a stronger antioxidant and anti‐inflammatory capacity than that from Sichuan (Zhao et al., 2017). Meanwhile, it has been reported that there existed differences in the quality and ingredients of *P*. *quinquefolius* L. from different origins (Chen et al., 2022). However, the potential chemical markers to discriminate American ginseng from different geographical origins have not been reported to date. Moreover, American ginseng roots vary in price depending on geographical origins. Therefore, it is necessary to discriminate American ginseng roots from different origins in the world.

Metabolomics is an omics technology focused on the comprehensive profiling of small‐molecular metabolites present in biological system through identification and quantification (Dudzik et al., 2018). As the final recipients of biological information, these metabolites can be considered as chemical phenotype of plants under specific environment (Gemperline et al., 2016). Therefore, metabolomics was widely used in the assessment of quality and geographical origin of plant products (Gika et al., 2014).

To find potential origin‐dependent markers of American ginseng from five different producing areas, including UC (America and Canada), HLJ (Heilongjiang province), JL (Jilin province), LN (Liaoning province), and SD (Shandong province), we established a metabolomics approach based on UPLC Q–Orbitrap MS to comprehensively analyze the chemical composition of American ginseng. Furthermore, multivariate statistical tools were used to investigate the differences in metabolites that could contribute to the geographical origin of American ginseng. The results will provide more foundation and reference for evaluation and discrimination of American ginseng.

## 2 MATERIALS AND METHODS

### 2.1 Plant materials

The samples for the discrimination of geographical origin were taken from five producing areas which were the main cultivating regions of American ginseng (America and Canada, Heilongjiang, Jilin, Liaoning, and Shandong in China). Each producing area selected 3–6 sampling points. The sampling points were scattered in each producing area, which can represent the main regions of American ginseng products. The sampling principle and design are described in detail in Supplementary Information Section 1 (Figure S1).

Nineteen origins of American ginseng were collected from five different producing areas in October 2021. Ninety individual plants were randomly harvested in each origin, and each 15 individual plants were mixed as one replication. All the voucher specimens were deposited in the laboratory of Institute of Special Animals and Plants, Chinese Academy of Agricultural Sciences, Changchun, China. Each sample's detailed information is given in Table S1.

### 2.2 Sample preparation

The samples were washed and dried at 38°C until a constant weight was achieved, then main roots were separated and pulverized into powder, and passed through a 60‐mesh sieve. A quantity of 0.1 g accurately weighed fine powder was extracted with 10 mL of 70% (v/v) methanol in an ultrasonic water bath for 60 min (Wang et al., 2019). The supernatant of extract was filtered through a syringe filter (0.22 μm) before liquid chromatography mass spectrometry system analysis. In addition, a quality control (QC) sample was prepared by pooling a 100‐μL aliquot of all test solutions.

### 2.3 HPLC Q–Orbitrap MS analysis

Chromatographic separation was performed with Ultimate 3000 UHPLC system (Thermo Fisher Scientific), equipped with an ACQUITY Premier HSS T3 column (1.8 μm, 100 × 2.1 mm). The column oven was maintained at 35°C. The mobile phases consisted of 0.1% formic acid in water (A) and 0.1% formic acid in acetonitrile (B). The mobile phase system was run in the following gradient program: 0–2 min, 0% B; 2–4 min, 0%–3% B; 4–6 min, 3%–15% B; 6–8 min, 15–25% B; 8–25 min, 25%–39% B; 25–32 min, 39%–60% B; 32–39 min, 60%–99% B; 39–41 min, 99% B; 41–42 min, 99%–0% B; 42–45 min, 0% B. The flow rate was 0.3 mL/min, and the injection volume was 2 μL.

High‐resolution MS data were recorded on an Orbitrap Fusion mass spectrometer (Thermo Fisher Scientific). Spray voltages were set at 3.7 kV and 2.7 kV for positive and negative modes, respectively. Other source parameters were set as follows: Ion Transfer Tube temperature, 320°C; Vaporizer temperature, 320°C; Sheath gas, 40 arbitrary units; Auxiliary gas, 5 arbitrary units. The Orbitrap analyzer scanned over a mass range of m/z 85–1500 at a resolution of 60,000 for MS^1^ scan and a resolution of 15,000 for HCD‐MS^2^ scan. The MS/MS product ions under mixed NCE (normalized collision energy) of 30%, 40%, and 55% were recorded to acquire more fragmentation information.

### 2.4 Data processing and metabolite identification

The acquired mass data were imported to Compound Discoverer 3.1 (Thermo Fisher Scientific) for peak detection and alignment. A data matrix, involving the information of the sample name, peak number (*t*_R_‐m/z pair) and normalized peak area, was finally obtained. Then, preprocessed data were imported into SIMCA‐14.1 (Umetrics) for PCA and OPLS‐DA analysis. Volcano plot was constructed on a cloud platform (https://cloud.metware.cn). Venn and heatmaps were generated by TBtools software (Chen et al., 2020). Statistical analyses were performed by one‐way ANOVA and LSD method using IBM SPSS Statistics 26 software (*p* < .01).

The saponins were identified by 14 standards and structural characterization according to the MS/MS spectrum (Shi et al., 2017; Wang et al., 2019; Yang et al., 2020, 2012; Zhang et al., 2020; Zuo et al., 2020), and the spectrum elucidation of representative compounds is shown in Supplementary Information Section 2 (Figures S2–S6). Other metabolites were recognized by 18 standards and mzCloud with match scores greater than 70, and the comparison MS/MS spectra of representative compounds between sample and mzCloud database are shown in Supplementary Information Section 3 (Figure S7).

## 3 RESULTS AND DISCUSSION

### 3.1 Metabolite profiling of American ginseng collected from different origins

**FIGURE 1:**
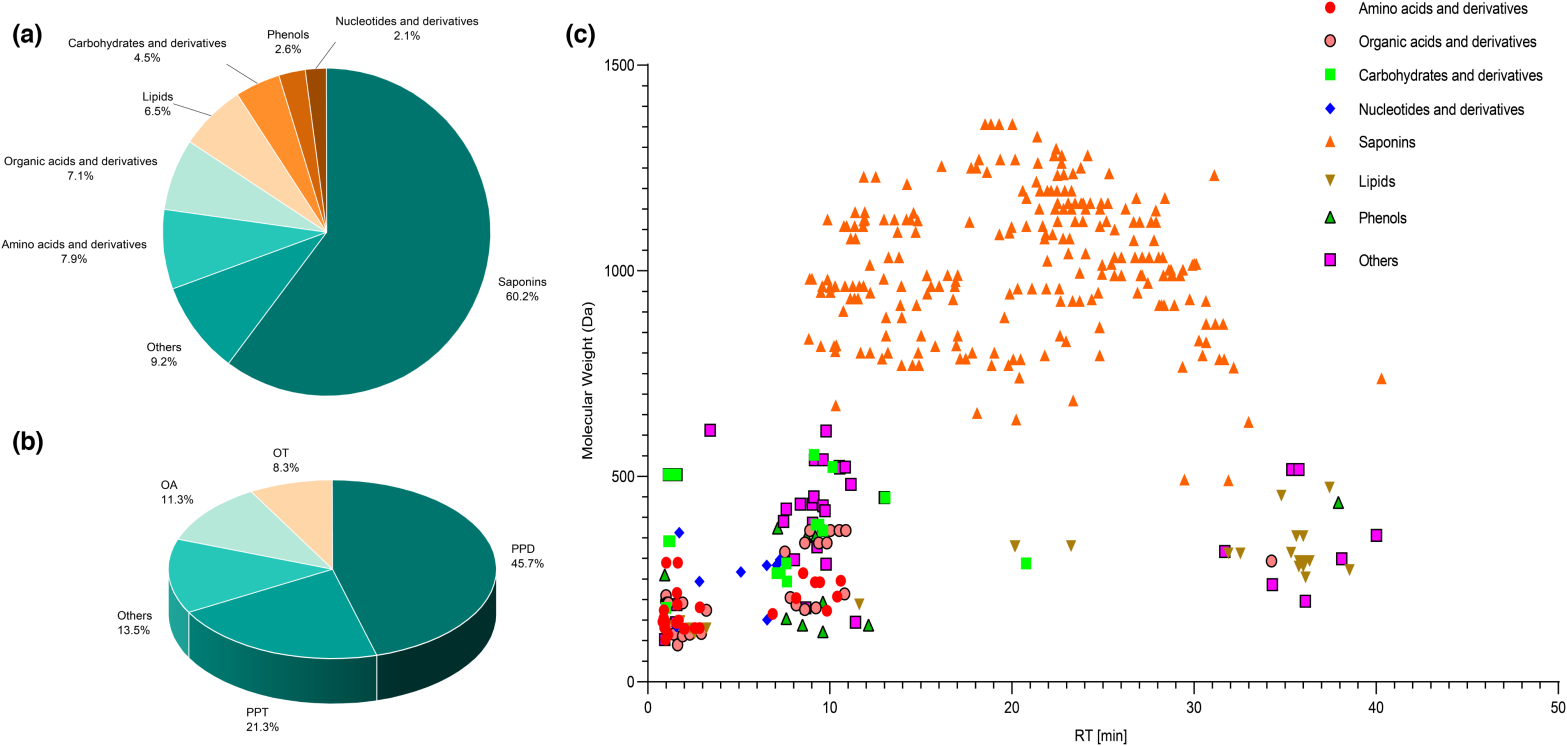
A general view of the metabolites identified in American ginseng samples (a) Classifications of the metabolites identified in the samples. (b) Classifications of the saponins identified in the samples. (c) The scatter plot of the retention time (RT) versus measured molecular weight of 382 metabolites identified.

A total of 3572 and 4009 spectra were detected from American ginseng in the negative and positive ion modes, respectively. Altogether 382 metabolites were identified in all samples (Table S2), including 230 saponins, 30 amino acids and derivatives, 27 organic acids and derivatives, 25 lipids, 17 carbohydrates and derivatives, 10 phenols, 8 nucleotides and derivatives, as well as 35 other metabolites (Figure 1a). As the most important secondary metabolites in American ginseng, saponins were divided into five subcategories, composed of protopanaxadiol, protopanaxatriol, oleanane, ocotillol, and other types, which accounted for 45.7%, 21.3%, 11.3%, 8.3%, and 13.5% of the identified saponins, respectively (Figure 1b). Furthermore, the retention time versus measured molecular weight of 382 metabolites was exhibited by a 2D scatter plot (Figure 1c). Overall, a large proportion of amino acids, organic acids, carbohydrates, phenols, and nucleotides were eluted before 14 min, most lipids were eluted after 32 min, and the elution times of saponins were between 8 and 35 min.

**FIGURE 2:**
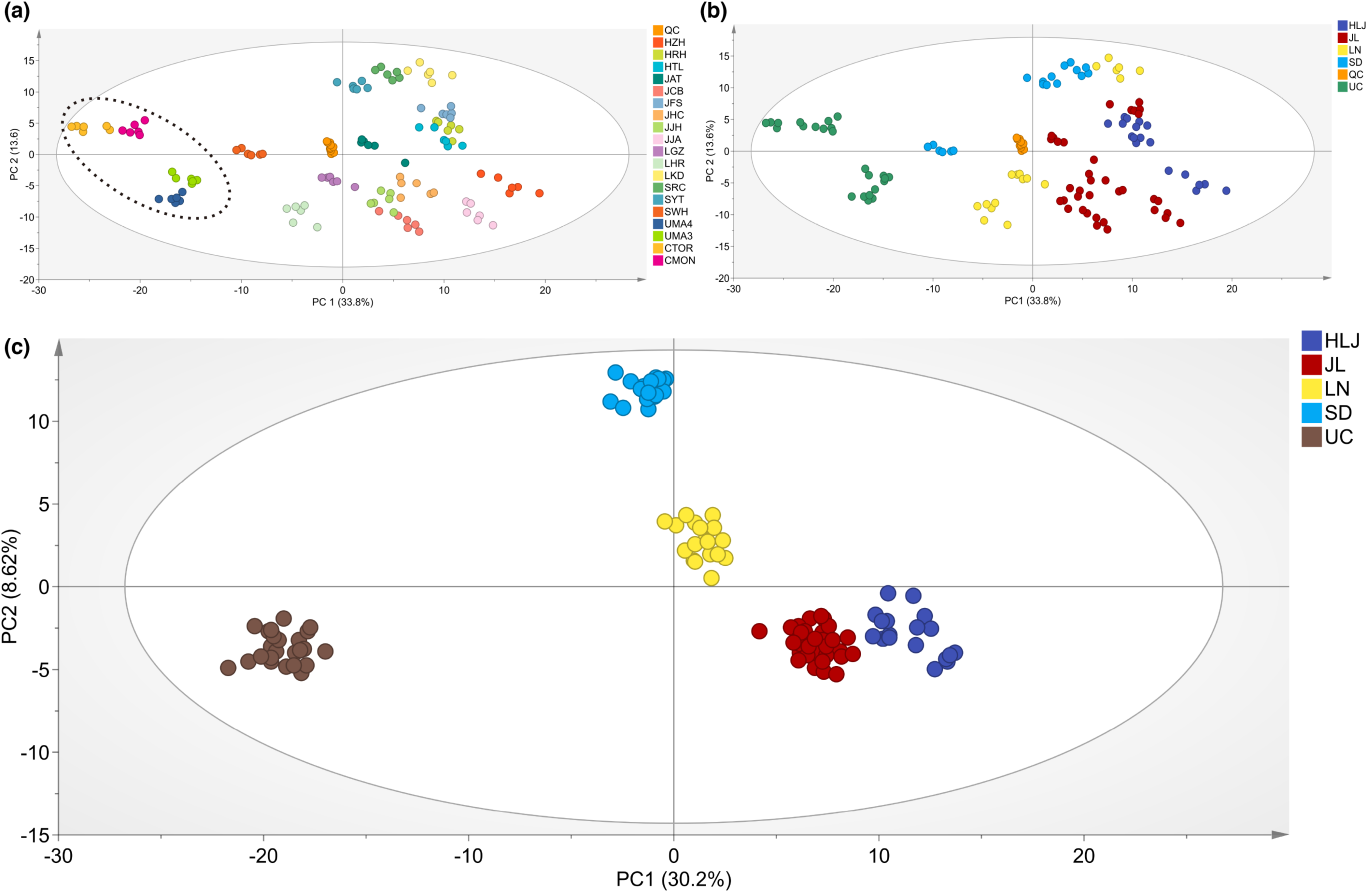
Multivariate statistical analysis. (a) PCA score plot of samples from different origins. (b) PCA score plot of samples from five producing areas. (c) OPLS‐DA score plot of samples from five producing areas.

The relative contents of these metabolites were used for principal component analysis (PCA). The score plot (Figure 2a) shows that QC samples used for quality control were tightly clustered, suggesting satisfactory stability of the system. The first two principal components explained 33.8% and 13.6% of the variability in the dataset, respectively. Samples within group were grouped together, indicating that samples from the same origin have similar chemical composition. Samples from UC were clearly separated and concentrated on the left side of the plot, which implied that the chemical constituents of UC samples are different from Chinese. Although Chinese samples from different producing areas were not clearly separated, there was a trend that samples from the same producing area grouped together (Figure 2b).

The orthogonal partial least squares discrimination analysis (OPLS‐DA) was a type of supervised classification tool, which can effectively eliminate irrelevant influences and screen for differential metabolites among comparison groups (Worley & Powers, 2016). Recent works showed that it was an effective tool to determinate geographical origins of medicinal plants (Mais et al., 2018; Wang et al., 2020). As shown in the OPLS‐DA score plot (Figure 2c), samples from five producing areas, including UC, HLJ, JL, LN, and SD, were easily classified. Data points with farther geographical distance were generally far away from each other in OPLS‐DA score plot, suggesting that geographical distance positively related to variation degree among five groups. As a closely related species of *P*. *quinquefolius* L., *Panax ginseng* from Gangwon, Gaeseong, Punggi, Chungbuk, Jeonbuk, and Anseong in Korean could also be differentiated by metabolites based on LC–MS data (Song et al., 2013), further demonstrating the impact of geographical location on metabolite constituents of *Panax* species. These geographical effects may be due to the varied climate and soil properties among different producing areas.

### 3.2 Differential metabolites of American ginseng cultivated from five producing areas

**FIGURE 3:**
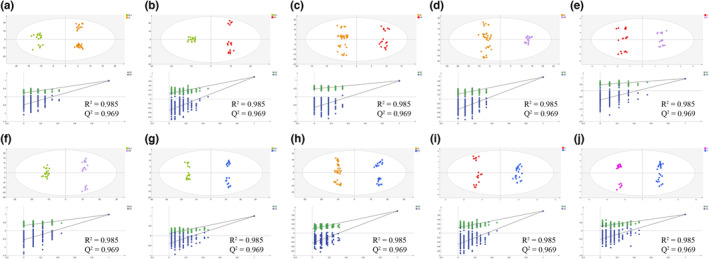
OPLS‐DA score plots and permutation tests in any two groups. (a) HLJ versus JL. (b) HLJ versus LN. (c) JL versus LN. (d) JL versus SD. (e) LN versus SD. (f) HLJ versus SD. (g) HLJ versus UC. (h) JL versus UC. (i) LN versus UC. (j) SD versus UC.

The VIP value reflects the importance of each metabolite in the OPLS‐DA model (Bylesjö et al., 2006). Firstly, the top three VIP metabolites were used to understand metabolite differences among five producing areas. Although there were certain differences in the contents of these three metabolites, they were indistinguishable among the five regions, making it difficult to clearly differentiate American ginseng from these regions (Figure S8). Thus, OPLS‐DA was further applied to evaluate the differences between each pairwise comparison. The R^2^Y and Q^2^Y values of the OPLS‐DA models were greater than 0.95 in any pairwise comparisons, confirming that the models had good explaining and prediction ability. In the permutation test (200 times), the original R^2^Y and Q^2^Y are always substantially higher than the corresponding permutation values, indicating the models were not overfitting (Figure 3). The results showed that samples from different producing areas were clearly separated in each pairwise comparison, which could, thus, be used to further identify differential metabolites.

**FIGURE 4:**
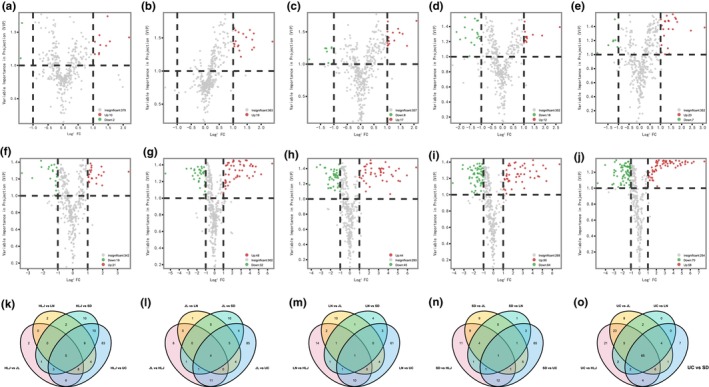
Screening of differential metabolites between different producing areas. Volcano plots for (a) LN versus SD, (b) LN versus JL, (c) HLJ versus JL, (d) HLJ versus LN, (e) SD versus JL, (f) HLJ versus SD, (g) LN versus UC, (h) SD versus UC, (i) JL versus UC, and (j) HLJ versus UC. Venn diagram analysis of shared differential metabolites of comparisons between (k) HLJ and other four regions, (l) JL and other four regions, (m) LN and other four regions, (n) SD and other four regions, (o) UC and other four regions.

Based on fold‐change (FC ≥2 or ≤0.5) and variable importance in projection (VIP≥1) score (Wang et al., 2020), 12 metabolites (10 upregulated, 2 downregulated) were identified as differential metabolites in LN versus SD, 19 metabolites (19 upregulated, 0 downregulated) in LN versus JL, 25 metabolites (17 upregulated, 8 downregulated) in HLJ versus JL, 30 metabolites (12 upregulated, 18 downregulated) in HLJ versus LN, 30 metabolites (23 upregulated, 7 downregulated) in SD versus JL, 40 metabolites (21 upregulated, 19 downregulated) in HLJ versus SD, 80 metabolites (48 upregulated, 32 downregulated) in LN versus UC, 89 metabolites (44 upregulated, 45 downregulated) in SD verssus UC, 114 metabolites (50 upregulated, 64 downregulated) in JL versus UC, 128 metabolites (58 upregulated, 70 downregulated) in HLJ versus UC (Figure 4A–J). Overall, the number of differential metabolites for UC was more than in Chinese producing areas. With respect to Chinese producing areas, neighboring provinces had fewer number of differential metabolites compared to nonadjacent provinces. These results show the tendency that the farther geographical distance is, the more difference will be.

Venn diagram showed the shared differential metabolites of comparisons between a certain producing area versus the other four (Figure 4K–O). A total of 65 shared differential metabolites were identified between UC and the other four producing areas, including 33 saponins, 9 lipids, 7 amino acids and derivatives, 6 nucleotides and derivatives, 3 organic acids and derivatives, 1 carbohydrate and derivatives, and 6 others (Table S3). However, there is less number of shared differential metabolites between any Chinese producing area and the other four. A total of five shared differential metabolites were identified between HLJ and the other four producing areas, including 4‐oxoproline (M44), leucine (M50), xanthosine (M61), 1,3,5‐trihydroxy‐4‐{[(2*E*)‐3‐(3‐hydroxy‐4‐methoxyphenyl)‐prop‐2‐enoyl] oxy} cyclohexane‐1‐carboxylic acid (M127), and PPD‐(Glc‐Glc)‐Glc‐Glc‐Butenoyl‐Mal (M261). Four shared differential metabolites were identified between JL and the other four producing areas, including Pseudo‐ginsenoside‐RT_5_ (M208) and three isomers of PPT‐(Glc‐Glc)‐Glc (M133, M147, and M152). Moreover, caffeic acid (M94) and PPD‐20‐Glc‐3‐Glc‐Glc‐Butenoyl (M341) was the only shared differential metabolites identified for LN and SD, respectively, compared with the other four producing areas.

**FIGURE 5:**
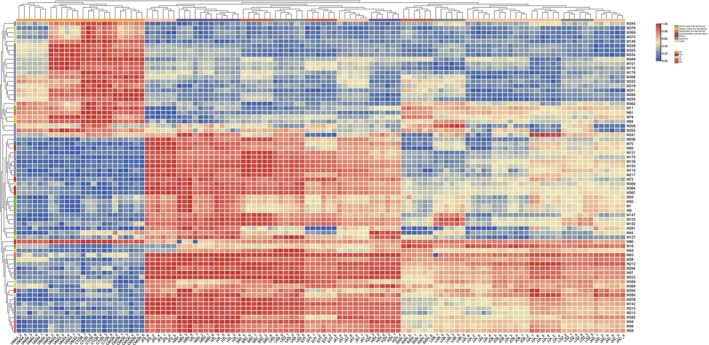
Heatmap hierarchical clustering analysis of shared differential metabolites.

The relative contents of these shared differential metabolites were used to perform a hierarchical cluster analysis (HCA). As shown in the heatmap (Figure 5), the samples from UC and China were classified into two main groups. It was noticeable that the contents of seven PPD‐type saponins with five glucose units were visibly lower in UC compared to any Chinese producing areas, including PPD‐(Glc‐Glc)‐Glc‐Glc‐Glc (M209), PPD‐(Glc‐Glc)‐Glc‐Glc‐Glc‐Mal (M210), PPD‐(Glc‐Glc)‐Glc‐Glc‐Glc‐Mal (M212), PPD‐(Glc‐Glc)‐Glc‐Glc‐Glc‐Mal (M215), PPD‐(Glc‐Glc)‐Glc‐Glc‐Glc (M217), PPD‐(Glc‐Glc)‐Glc‐Glc‐Glc‐Mal (M223), and PPD‐(Glc‐Glc)‐Glc‐Glc‐Glc (M226). They were the only seven saponins that carry five glucose units in American ginseng. On the contrary, the contents of seven PPD‐type saponins with four glucose units were visibly higher in UC than the other four Chinese producing areas. The seven saponins were PPD‐(Glc‐Glc)‐Glc‐Glc‐Mal (M232), PPD‐(Glc‐Glc)‐Glc‐Glc‐Ac (M252), PPD‐(Glc‐Glc)‐Glc‐Glc‐Ac (M286), PPD‐(Glc‐Glc)‐Glc‐Glc‐Ac (M295), PPD‐(Glc‐Glc)‐Glc‐Glc‐Mal‐Ac (M301), PPD‐(Glc‐Glc)‐Glc‐Glc‐Ac (M308), and PPD‐20‐Glc‐Glc‐3‐Glc‐Glc‐Octenoyl (M349). A similar result was also observed in previous report about American ginseng from 10 different origins. Content of Rg_1_ (PPT‐20‐Glc‐6‐Glc) with two sugar units and Re (PPT‐20‐Glc‐6‐Glc‐Rha) with three sugar units was visibly lower in four origins compared to other six (Schlag & McIntosh, 2006). These results indicated that location (growing environment) may affect the transfer of sugar units of saponins.

### 3.3 Discovery of potential markers to distinguish American ginseng from different producing areas

**FIGURE 6:**
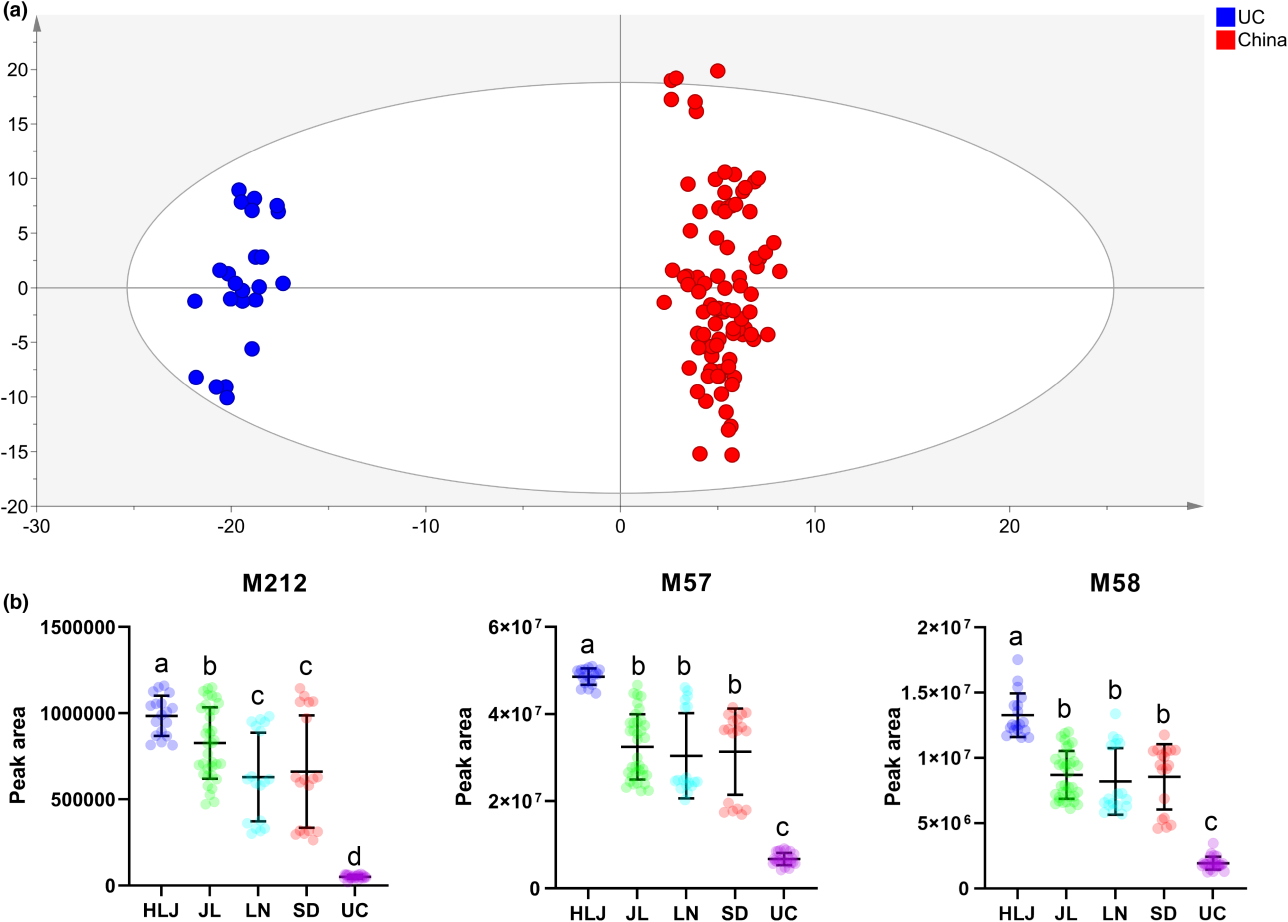
(a) OPLS‐DA score plots of American ginseng from UC and China. (b) The relative content of the top three metabolites ranked by VIP score in OPLS‐DA from UC versus China. Different letter indicates significant differences between different producing areas (*p* < .01).

Based on the results of HCA (Figure 5), only UC samples could clearly be separated from all other producing areas, while samples from four provinces in China could not be separated from each other. Therefore, potential markers to distinguish American ginseng between UC and China were screened in the first step. To find more influential metabolites among shared differential metabolites between UC and China, OPLS‐DA models were established for samples from the two farther producing areas (Figure 6a). Based on VIP ranking, PPD‐(Glc‐Glc)‐Glc‐Glc‐Glc‐Mal (M212), guanosine (M57), and guanine (M58) with top three VIP scores, were identified as the potential markers. The relative content of these three markers in US samples was significantly lower than that of China (Figure 6b). Chen et al. (2022) reported that ginsenoside F_2_, ginsenoside Rg_5_, pseudo‐ginsenoside F_11_, and quinquenoside R_1_ were the typical compounds for distinguishing Wisconsin American ginseng (USA) samples from other areas. Among them, quinquenoside R_1_ (PPD‐Glc‐Glc‐Glc‐Glc‐Ac) with four glucose units had higher content in USA samples in both Chen et al. and our work. However, quinquenoside R_1_ was not gained very high VIP score in our study. Compared with Chen's results, three new chemical markers were found for the discrimination of American ginseng between UC and China.

**FIGURE 7:**
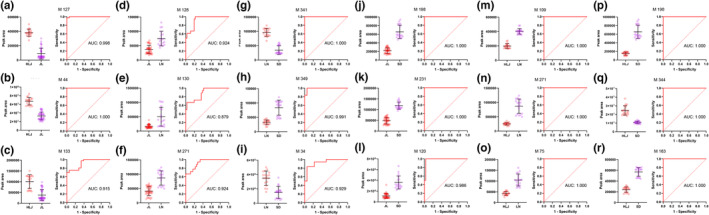
Relative content and ROC curves of the top three metabolites ranked by VIP value in OPLS‐DA from (a–c) HLJ versus JL, (d–f) JL versus LN, (g–i) LN versus SD, (j–l) JL versus SD, (m–o) HLJ versus LN, (p–r) HLJ versus SD.

In the second step, potential markers were screened to distinguish four producing areas in China. Firstly, the top three metabolites ranked by VIP value in OPLS‐DA in each pairwise comparison were selected as candidates. Then, receiver operating characteristics (ROC) analysis was used to evaluate prediction accuracy. Ultimately, based on the criteria for the area under the curve (AUC) value over 0.9 which provided excellent prediction accuracy (Xia et al., 2013), 1,3,5‐trihydroxy‐4‐{[(2*E*)‐3‐(3‐hydroxy‐4‐methoxyphenyl)prop‐2‐enoyl]oxy}cyclohexane‐1‐carboxylic acid (M127), 4‐Oxoproline (M44), and PPT‐(Glc‐Glc)‐Glc (M133) were identified as potential markers for discrimination of Heilongjiang and Jilin samples (Figure 7A–C). Similarly, 4‐[4‐(4‐Hydroxy‐3‐methoxyphenyl) tetrahydro‐1H,3H‐furo[3,4‐c] furan‐1‐yl]‐2‐methoxyphenyl (M128) and (15*Z*)‐9,12,13‐Trihydroxy‐15‐octadecenoic acid (M271) were identified as potential markers for discrimination of Jilin and Liaoning samples (Figure 7D–F). PPD‐20‐Glc‐3‐Glc‐Glc‐Butenoyl (M341), PPD‐20‐Glc‐Glc‐3‐Glc‐Glc‐Octenoyl (M349), and Acetyl arginine (M34) were identified for discrimination of Liaoning and Shandong samples (Figure 7G–I). 457‐Glc‐Glc‐Rha (M198), 20(R)‐Ginsenoside Rg_2_ (M231), and 491‐20‐Glc‐6‐Glc (M120) were identified for discrimination of Jilin and Shandong samples (Figure 7J–L). (2S,3R,4S,5R)‐2‐{[(2R,3R,4S,5S,6R)‐4,5‐dihydroxy‐6‐(hydroxymethyl)‐2‐(2‐phenylethoxy)oxan‐3‐yl]oxy}oxane‐3,4,5‐triol (M109), (15Z)‐9,12,13‐Trihydroxy‐15‐octadecenoic acid (M271), and 2‐(4‐Hydroxyphenyl)ethyl 6‐O‐[(2R,3R,4R)‐3,4‐dihydroxy‐4‐(hydroxymethyl)tetrahydro‐2‐furanyl]‐beta‐D‐glucopyranoside (M75) were identified for discrimination of Heilongjiang and Liaoning samples (Figure 7M–O). 457‐Glc‐Glc‐Rha (M198), Ginsenoside F_2_ (M344), and PPT‐(Glc‐Glc)‐Glc‐Glc (M163) were identified for discrimination of Heilongjiang and Shandong samples (Figure 7P–R).

It was reported that the content of 20(S)‐Ginsenoside Rg_2_ in American ginseng from northeast is higher than that of Shandong province in China (Huang et al., 2013), which was in accordance with our observation. But it is interesting to note that 20(*R*)‐Ginsenoside Rg_2_ makes a more obvious distinction between the two regions in our study. Si (2021) screened 19 differential metabolites between American ginseng from Jilin and Shandong in China; however, none of them was differential metabolite in our study. To sum up, the study in this part provided 17 potential markers for distinguishing American ginseng from four producing areas in China and provided over 91% of correct predictions. As far as we know, the 17 potential markers have not been reported.

## 4 CONCLUSION

In this study, a metabolomic method based on the UPLC–Orbitrap fusion platform was established to comprehensively determine and analyze metabolites of American ginseng. Totally, 382 metabolites were identified in American ginseng from five producing areas. There are larger number of differential metabolites between UC and Chinese producing areas than within four Chinese producing areas. Twenty potential chemical markers were identified for the first time, which could effectively discriminate (ROC >91%) American ginseng from different producing areas. The results provide new insights into the metabolite composition of American ginseng from different origins, which greatly benefits the quality control and geographical origins discrimination of American ginseng.

## AUTHOR CONTRIBUTIONS

**Shifeng Pang:** Data curation (lead); investigation (lead); software (lead); visualization (lead); writing – original draft (lead); writing – review and editing (lead). **Xiangmin Piao:** Data curation (equal); software (equal). **Xiaohao Zhang:** Writing – review and editing (equal). **Xiaolin Chen:** Data curation (supporting); visualization (supporting); writing – review and editing (supporting). **Hao Zhang:** Project administration (equal); supervision (equal); writing – review and editing (equal). **Yinping Jin:** Writing – review and editing (equal). **Zheng Li:** Software (equal); visualization (equal); writing – review and editing (equal). **Yingping Wang:** Project administration (equal); resources (lead); supervision (lead); writing – review and editing (equal).

## FUNDING INFORMATION

This work was supported by grants from the Science and Technology Development Project of Jilin Province, Grant/Award Number: 20230505028ZP; the Scientific and Technological Innovation Project of the Chinese Academy of Agricultural Science, Grant/Award Number: CAAS‐ASTIP‐ISAPS‐2021‐ISAPS; and the National Key Research and Development Program of China, Grant/Award Number: 2021YFD1600901.

## CONFLICT OF INTEREST STATEMENT

The authors declare that there is no conflict of interest.

## ETHICAL STATEMENT

This study does not involve any human or animal testing.

## Supporting information


Table S1

Table S2

Table S3

Appendix S1

## Data Availability

Research data are not shared.
