# Supplementary material for: Discrimination for geographical origin of Panax quinquefolius L. using UPLC Q‐Orbitrap MS‐based metabolomics approach
Source: Food Sci Nutr. 2023 Jul 10;11(8):4843–52. doi: 10.1002/fsn3.3461 (PMC10420767; doi:10.1002/fsn3.3461)
Supplement: Supplementary file 1 — Table S1 [file FSN3-11-4843-s001.docx]

Table S1 Details of American ginseng samples

| Code No. | Sources | Age (year) | Latitude | Longitude |
| --- | --- | --- | --- | --- |
| HZH_1-6 | Zhanhe, wudalianchi，Heilongjiang | 3 | 48°41′ N | 126°59′ E |
| HRH_1-6 | Raohe, Shuangyashan，Heilongjiang | 3 | 47°20′ N | 133°56′ E |
| HTL_1-6 | Tieli, Yichun，Heilongjiang | 4 | 47°10′ N | 128°20′ E |
| JAT_1-6 | Antu，Jilin | 4 | 43°15′ N | 128°52′ E |
| JHC_1-6 | Hunchun，Jilin | 4 | 42°53′ N | 130°40′ E |
| JJH_1-6 | Jiaohe，Jilin | 3 | 43°42′ N | 127°39′ E |
| JFS_1-6 | Fusong，Jilin | 3 | 42°22′ N | 127°23′ E |
| JCB_1-6 | Changbai，Jilin | 3 | 41°26′ N | 128°1′ E |
| JJA_1-6 | Ji′an，Jilin | 3 | 41°25′ N | 125°57′ E |
| LHR_1-6 | Huanren，Liaoning | 4 | 41°13′ N | 124°54′ E |
| LKD_1-6 | Kuandian，Liaoning | 3 | 40°40′ N | 124°39′ E |
| LGZ_1-6 | Gaizhou，Liaoning | 4 | 40°16′ N | 122°30′ E |
| SYT_1-6 | Yantai，Shandong | 3 | 37°31′ N | 121°25′ E |
| SRC_1-6 | Rongcheng，Shandong | 4 | 37°13′ N | 122°30′ E |
| SWH_1-6 | Weihai，Shandong | 4 | 37°60′ N | 122°70′ E |
| CTOR_1-6 | Toronto，Canada | 4 | 43°55′ N | 79°15′ W |
| CMON_1-6 | Montreal，Canada | 4 | 45°38′ N | 74°11′ W |
| UMA3_1-6 | Marathon，America | 3 | 45°00′ N | 89°58′ W |
| UMA4_1-6 | Marathon，America | 4 | 44°93′ N | 89°82′ W |
