# Supplementary material for: Discrimination for geographical origin of Panax quinquefolius L. using UPLC Q‐Orbitrap MS‐based metabolomics approach
Source: Food Sci Nutr. 2023 Jul 10;11(8):4843–52. doi: 10.1002/fsn3.3461 (PMC10420767; doi:10.1002/fsn3.3461)
Supplement: Supplementary file 2 — Table S2 [file FSN3-11-4843-s004.docx]

Table S2 Detailed information of the 382 compounds in American ginseng

| No. | RT [min] | Theoretical Mass (Da) | Measured Mass (Da) | Formula | Error  [ppm] | Ionization  model | Identification compound | Fragment ion (MS/MS) | Class Ⅰ | Class Ⅱ |
| --- | --- | --- | --- | --- | --- | --- | --- | --- | --- | --- |
| M1 | 0.82 | 146.10553 | 146.10592 | C6 H14 N2 O2 | 2.67 | [M+H]^+^ | Lysine* | 130.0888,84.0809 | Amino acids and derivatives | Amino acids and derivatives |
| M2 | 0.87 | 155.06948 | 155.0701 | C6 H9 N3 O2 | 4.00 | [M+H]^+^ | Histidine* | 156.0771,110.0715,93.0449,83.0605 | Amino acids and derivatives | Amino acids and derivatives |
| M3 | 0.88 | 174.11168 | 174.11087 | C6 H14 N4 O2 | -4.65 | [M-H]^-^ | Arginine* | 131.08151,114.0550 | Amino acids and derivatives | Amino acids and derivatives |
| M4 | 0.91 | 132.05349 | 132.05409 | C4 H8 N2 O3 | 4.54 | [M+H]^+^ | Asparagine* | 133.0611,116.0344,74.0238 | Amino acids and derivatives | Amino acids and derivatives |
| M5 | 0.93 | 103.09971 | 103.10013 | C5 H13 N O | 4.07 | [M+H]^+^ | Choline | 104.1073,60.0809,58.06532 | Others | Others |
| M6 | 0.93 | 133.03751 | 133.03697 | C4 H7 N O4 | -4.06 | [M-H]^-^ | Aspartic acid* | 115.0025,88.0039,72.0081 | Amino acids and derivatives | Amino acids and derivatives |
| M7 | 0.93 | 260.02972 | 260.0287 | C6 H13 O9 P | -3.92 | [M-H]^-^ | α-D-Mannose 1-phosphate | 241.0103,138.9789,78.9581 | Phenols | Phenols |
| M8 | 0.93 | 146.06914 | 146.06972 | C5 H10 N2 O3 | 3.97 | [M+H]^+^ | DL-Glutamine | 147.0771,130.0503,84.0446 | Amino acids and derivatives | Amino acids and derivatives |
| M9 | 0.95 | 146.06914 | 146.06867 | C5 H10 N2 O3 | -3.22 | [M-H]^-^ | D-(-)-Glutamine | 127.0502,109.0397,84.0446,74.0239 | Amino acids and derivatives | Amino acids and derivatives |
| M10 | 0.97 | 129.04259 | 129.04216 | C5 H7 N O3 | -3.33 | [M-H]^-^ | 4-Oxoproline | 128.0341,84.8291 | Amino acids and derivatives | Amino acids and derivatives |
| M11 | 0.97 | 147.05316 | 147.05377 | C5 H9 N O4 | 4.15 | [M+H]^+^ | Glutamic acid* | 130.0502,84.0446,56.0496 | Amino acids and derivatives | Amino acids and derivatives |
| M12 | 0.98 | 103.06333 | 103.06356 | C4 H9 N O2 | 2.23 | [M+H]^+^ | Υ-Aminobutyric acid (GABA) | 104.0709,87.0442,69.0336,60.0809 | Amino acids and derivatives | Amino acids and derivatives |
| M13 | 0.99 | 196.0583 | 196.05733 | C6 H12 O7 | -4.95 | [M-H]^-^ | Gluconic acid | 177.0393,159.0288,129.0183,75.0079 | Organic acids and derivatives | Organic acids and derivatives |
| M14 | 1.00 | 180.06339 | 180.06389 | C6 H12 O6 | 2.78 | [M+FA-H]^-^ | D-(-)-Glucose | 119.0339,89.0234,59.0148 | Carbohydrates and derivatives | Carbohydrates and derivatives |
| M15 | 1.00 | 210.03757 | 210.03657 | C6 H10 O8 | -4.76 | [M-H]^-^ | D-Saccharic acid | 191.01848,133.0133,85.0287 | Organic acids and derivatives | Organic acids and derivatives |
| M16 | 1.01 | 290.12263 | 290.12372 | C10 H18 N4 O6 | 3.76 | [M+H]^+^ | Argininosuccinic acid | 246.1091,116.0709,70.0653 | Amino acids and derivatives | Amino acids and derivatives |
| M17 | 1.05 | 192.027 | 192.02615 | C6 H8 O7 | -4.43 | [M-H]^-^ | Isocitric acid | 147.0285,111.0076,85.0286 | Organic acids and derivatives | Organic acids and derivatives |
| M18 | 1.06 | 136.03717 | 136.03668 | C4 H8 O5 | -3.60 | [M-H]^-^ | L-Threonic acid | 117.0184,89.0234,75.0079,71019 | Lipids | Fatty acids and derivatives |
| M19 | 1.12 | 115.06333 | 115.06377 | C5 H9 N O2 | 3.82 | [M+H]^+^ | D-(+)-Proline* | 98.0604,70.0653 | Amino acids and derivatives | Amino acids and derivatives |
| M20 | 1.12 | 504.16903 | 504.17093 | C18 H32 O16 | 3.77 | [M+NH_4_]^+^ | D-Raffinose | 325.1149,145.0499,127.0393,91.0390,85.0286 | Carbohydrates and derivatives | Carbohydrates and derivatives |
| M21 | 1.12 | 126.03169 | 126.0322 | C6 H6 O3 | 4.05 | [M+H]^+^ | 5-Hydroxymethyl-2-furaldehyde | 109.0285,81.0336,69.0336,53.0387 | Others | Alcohols |
| M22 | 1.13 | 192.06339 | 192.06252 | C7 H12 O6 | -4.53 | [M-H]^-^ | D-(-)-Quinic acid | 191.0551,173.0445,127.0391,85.0287 | Organic acids and derivatives | Organic acids and derivatives |
| M23 | 1.13 | 342.11621 | 342.11777 | C12 H22 O11 | 4.56 | [M+NH_4_]^+^ | sucrose | 145.0501,127.0394,97.0287,85.0286 | Carbohydrates and derivatives | Carbohydrates and derivatives |
| M24 | 1.14 | 504.16316 | 504.16438 | C25 H28 O11 | 2.42 | [M+FA-H]^-^ | 3-[2-(1,3-Benzodioxol-5-yl)-7-methoxy-1-benzofuran-5-yl]-3-hydroxypropyl hexopyranoside | 503.1596,341.1070,179.0548,101.0244,89.0235 | Others | Others |
| M25 | 1.23 | 342.11621 | 342.11501 | C12 H22 O11 | -3.51 | [M+FA-H]^-^ | D-(+)-Maltose | 341.1076,281.0865,221.0656,179.0550,101.0236,71.0131 | Carbohydrates and derivatives | Carbohydrates and derivatives |
| M26 | 1.40 | 134.02152 | 134.02089 | C4 H6 O5 | -4.70 | [M-H]^-^ | Malic acid* | 133.0132,115.0027,89.0236,71.0131 | Organic acids and derivatives | Organic acids and derivatives |
| M27 | 1.42 | 116.01096 | 116.01073 | C4 H4 O4 | -1.98 | [M-H]^-^ | Fumaric acid | 71.0129 | Organic acids and derivatives | Organic acids and derivatives |
| M28 | 1.54 | 144.04226 | 144.04218 | C6 H8 O4 | -0.56 | [M-H_2_O+H]^+^ | 5-(hydroxymethyl)-4-methoxy-2,5-dihydrofuran-2-one | 109.0286,81.0337,53.0387 | Others | Others |
| M29 | 1.56 | 504.16903 | 504.17089 | C18 H32 O16 | 3.69 | [M+H]^+^ | D-Raffinose | 163.0603,145.0498,127.0392,85.0285 | Carbohydrates and derivatives | Carbohydrates and derivatives |
| M30 | 1.56 | 143.09463 | 143.09514 | C7 H13 N O2 | 3.56 | [M+H]^+^ | 1-Aminocyclohexanecarboxylic acid | 144.1022,98.0966 | Amino acids and derivatives | Amino acids and derivatives |
| M31 | 1.57 | 135.0545 | 135.05499 | C5 H5 N5 | 3.63 | [M+H]^+^ | Adenine | 136.0620,119.0354 | Nucleotides and derivatives | purines |
| M32 | 1.59 | 188.07971 | 188.08018 | C7 H12 N2 O4 | 2.50 | [M+H]^+^ | methyl 2-(acetylamino)-4-amino-4-oxobutanoate | 130.05009,84.0445 | Others | Others |
| M33 | 1.60 | 504.16316 | 504.16538 | C25 H28 O11 | 4.40 | [M+FA-H]^-^ | 3-[2-(1,3-Benzodioxol-5-yl)-7-methoxy-1-benzofuran-5-yl]-3-hydroxypropyl hexopyranoside | 503.1594,341.1071,179.0547,101.0235 | Others | Others |
| M34 | 1.60 | 216.12224 | 216.12274 | C8 H16 N4 O3 | 2.31 | [M+H]^+^ | Acetylarginine | 175.1195,158.0816,116.0709,70.0653 | Amino acids and derivatives | Amino acids and derivatives |
| M35 | 1.62 | 188.07971 | 188.07894 | C7 H12 N2 O4 | -4.09 | [M-H]^-^ | N-Acetyl-L-glutamine | 145.0606,127.0501,101.0711,84.0446,58.0291 | Amino acids and derivatives | Amino acids and derivatives |
| M36 | 1.64 | 90.03169 | 90.03135 | C3 H6 O3 | -3.78 | [M-H]^-^ | L-(+)-Lactic acid* | 89.0235,87.0079,71.0130 | Organic acids and derivatives | Organic acids and derivatives |
| M37 | 1.65 | 290.12263 | 290.12354 | C10 H18 N4 O6 | 3.14 | [M+H]^+^ | Argininosuccinic acid | 175.1193,116.0708,70.0652 | Amino acids and derivatives | Amino acids and derivatives |
| M38 | 1.69 | 149.05105 | 149.05148 | C5 H11 N O2 S | 2.88 | [M+H]^+^ | L-(-)-Methionine* | 133.0320,104.0530,61.0108 | Amino acids and derivatives | Amino acids and derivatives |
| M39 | 1.73 | 363.058 | 363.05678 | C10 H14 N5 O8 P | -3.36 | [M-H]^-^ | Guanosine monophosphate (GMP) | 362.0491,211.0001,78.9582 | Nucleotides and derivatives | Ribonucleoside |
| M40 | 1.78 | 148.03717 | 148.03687 | C5 H8 O5 | -2.03 | [M-H]^-^ | D-α-Hydroxyglutaric acid | 129.0183,103.0391,85.0286 | Lipids | Fatty acids and derivatives |
| M41 | 1.91 | 112.01604 | 112.01574 | C5 H4 O3 | -2.68 | [M-H]^-^ | 2-Furoic acid | 111.0079,67.018 | Organic acids and derivatives | Organic acids and derivatives |
| M42 | 1.91 | 192.027 | 192.02606 | C6 H8 O7 | -4.90 | [M-H]^-^ | Citric acid* | 173.0081,129.0184,111.0079,87.0080 | Organic acids and derivatives | Organic acids and derivatives |
| M43 | 1.98 | 129.04259 | 129.0431 | C5 H7 N O3 | 3.95 | [M+H]^+^ | L-Pyroglutamic acid | 102.0549,84.0445,56.0496 | Amino acids and derivatives | Amino acids and derivatives |
| M44 | 2.02 | 129.04259 | 129.04211 | C5 H7 N O3 | -3.72 | [M-H]^-^ | 4-Oxoproline | 128.0342,84.0446,82.0290 | Amino acids and derivatives | Amino acids and derivatives |
| M45 | 2.30 | 116.01096 | 116.01046 | C4 H4 O4 | -4.31 | [M-H]^-^ | Fumaric acid* | 115.0027,71.0130 | Organic acids and derivatives | Organic acids and derivatives |
| M46 | 2.32 | 130.02661 | 130.02613 | C5 H6 O4 | -3.69 | [M-H]^-^ | Citraconic acid | 85.0295 | Lipids | Fatty acids and derivatives |
| M47 | 2.57 | 118.02661 | 118.02622 | C4 H6 O4 | -3.30 | [M-H]^-^ | Succinic acid | 117.0189,99.0178,73.0287 | Lipids | Fatty acids and derivatives |
| M48 | 2.58 | 131.09463 | 131.09497 | C6 H13 N O2 | 2.59 | [M+H]^+^ | Isoleucine* | 86.09654,69.0670 | Amino acids and derivatives | Amino acids and derivatives |
| M49 | 2.83 | 244.06954 | 244.06896 | C9 H12 N2 O6 | -2.38 | [M-H]^-^ | Uridine | 200.0549,110.0237,82.0289 | Nucleotides and derivatives | Ribonucleoside |
| M50 | 2.84 | 131.09463 | 131.09497 | C6 H13 N O2 | 2.59 | [M+H]^+^ | leucine* | 86.0965,69.0699 | Amino acids and derivatives | Amino acids and derivatives |
| M51 | 2.86 | 181.07389 | 181.0743 | C9 H11 N O3 | 2.26 | [M+H]^+^ | L-Tyrosine* | 165.0551,136.0760,123.0443,119.0494,95.0493,91.0544 | Amino acids and derivatives | Amino acids and derivatives |
| M52 | 2.94 | 118.02661 | 118.02612 | C4 H6 O4 | -4.15 | [M-H]^-^ | Methylmalonic acid | 73.0287,55.0182 | Organic acids and derivatives | Organic acids and derivatives |
| M53 | 3.22 | 130.02661 | 130.02598 | C5 H6 O4 | -4.85 | [M-H]^-^ | Citraconic acid | 85.0286 | Lipids | Fatty acids and derivatives |
| M54 | 3.22 | 174.01644 | 174.016 | C6 H6 O6 | -2.53 | [M-H]^-^ | 1,2,3-cyclopropanetricarboxylic acid | 129.0182,111.0078,85.0286 | Organic acids and derivatives | Organic acids and derivatives |
| M55 | 3.43 | 612.15196 | 612.15102 | C20 H32 N6 O12 S2 | -1.54 | [M-H]^-^ | L-Glutathione oxidized | 304.0594,272.0873,143.0449 | Others | Organonitrogens |
| M56 | 5.11 | 267.09675 | 267.09722 | C10 H13 N5 O4 | 1.76 | [M+H]^+^ | Adenosine | 136.0620,85.0286 | Nucleotides and derivatives | Ribonucleoside |
| M57 | 6.53 | 283.09167 | 283.09111 | C10 H13 N5 O5 | -1.98 | [M-H]^-^ | Guanosine | 192.0517,150.0409,107.0354 | Nucleotides and derivatives | Ribonucleoside |
| M58 | 6.55 | 151.04941 | 151.04977 | C5 H5 N5 O | 2.38 | [M+H]^+^ | Guanine | 152.0571,135.0305,110.0352 | Nucleotides and derivatives | purines |
| M59 | 6.85 | 165.07898 | 165.07937 | C9 H11 N O2 | 2.36 | [M+H]^+^ | L-Phenylalanine* | 120.0809,103.0544 | Amino acids and derivatives | Amino acids and derivatives |
| M60 | 7.07 | 264.1209 | 264.12165 | C11 H20 O7 | 2.84 | [M+FA-H]^-^ | 2-Hydroxy-2-methyl-3-buten-1-yl beta-D-glucopyranoside | 146.0809,71.0129,59.0131 | Carbohydrates and derivatives | Carbohydrates and derivatives |
| M61 | 7.08 | 284.07568 | 284.07466 | C10 H12 N4 O6 | -3.59 | [M-H]^-^ | Xanthosine | 283.0668,151.0249 | Nucleotides and derivatives | Ribonucleoside |
| M62 | 7.13 | 374.1213 | 374.12129 | C16 H22 O10 | -0.03 | [M+FA-H]^-^ | 1-O-(3,4,5-Trimethoxybenzoyl)-beta-L-galactopyranose | 211.0596,153.0543 | Phenols | Phenols |
| M63 | 7.25 | 297.10732 | 297.10687 | C11 H15 N5 O5 | -1.51 | [M-H]^-^ | 2'-O-Methylguanosine | 191.0438,150.0412,107.0354 | Nucleotides and derivatives | Ribonucleoside |
| M64 | 7.28 | 264.1209 | 264.12175 | C11 H20 O7 | 3.22 | [M+FA-H]^-^ | 2-Hydroxy-2-methyl-3-buten-1-yl beta-D-glucopyranoside | 113.0233,71.0130,59.0131 | Carbohydrates and derivatives | Carbohydrates and derivatives |
| M65 | 7.45 | 390.1526 | 390.15352 | C17 H26 O10 | 2.36 | [M+NH_4_]^+^ | methyl (1S)-6-hydroxy-7-methyl-1-{[(2S,3R,4S,5S,6R)-3,4,5-trihydroxy-6-(hydroxymethyl)oxan-2-yl]oxy}-1H,4aH,5H,6H,7H,7aH-cyclopenta[c]pyran-4-carboxylate | 211.0968,179.0709,137.0599 | Others | Others |
| M66 | 7.52 | 316.07943 | 316.07864 | C13 H16 O9 | -2.50 | [M-H]^-^ | N-Acetyl-L-phenylatanine | 152.0102,108.0206 | Organic acids and derivatives | Organic acids and derivatives |
| M67 | 7.61 | 154.02661 | 154.02602 | C7 H6 O4 | -3.83 | [M-H]^-^ | Gentisic acid | 109.0285,108.0207,81.0338 | Phenols | Phenols |
| M68 | 7.61 | 420.12678 | 420.12769 | C17 H24 O12 | 2.17 | [M+H]^+^ | 3-{[(2S,3R,4S,5S,6R)-6-({[(2R,3R,4R)-3,4-dihydroxy-4-(hydroxymethyl)oxolan-2-yl]oxy}methyl)-3,4,5-trihydroxyoxan-2-yl]oxy}-2-methyl-4H-pyran-4-one | 127.0392,85.0285 | Others | Others |
| M69 | 7.61 | 288.08452 | 288.08512 | C12 H16 O8 | 2.08 | [M+H]^+^ | Maltol-Glucoside | 127.0392 | Carbohydrates and derivatives | Carbohydrates and derivatives |
| M70 | 7.64 | 244.16746 | 244.16704 | C13 H24 O4 | -1.72 | [M+Cl]^-^ | 4-(Hydroxymethyl)-3-(1-hydroxyoctyl)dihydro-2(3H)-furanone | 279.1333 | Carbohydrates and derivatives | Carbohydrates and derivatives |
| M71 | 7.83 | 205.03751 | 205.03713 | C10 H7 N O4 | -1.85 | [M-H]^-^ | Xanthurenic acid | 160.0389,159.0311,118.0287 | Organic acids and derivatives | Organic acids and derivatives |
| M72 | 8.05 | 297.08956 | 297.09026 | C11 H15 N5 O3 S | 2.36 | [M+H]^+^ | 5'-S-Methyl-5'-thioadenosine | 163.0432,136.0620,119.0354 | Others | Others |
| M73 | 8.15 | 204.08988 | 204.09043 | C11 H12 N2 O2 | 2.69 | [M+H]^+^ | DL-Tryptophan* | 188.0712,146.0605,118.0655 | Amino acids and derivatives | Amino acids and derivatives |
| M74 | 8.15 | 187.06333 | 187.06381 | C11 H9 N O2 | 2.57 | [M+H]^+^ | Indole-3-acrylic acid | 146.0605,118.0654,91.0544 | Organic acids and derivatives | Organic acids and derivatives |
| M75 | 8.38 | 432.16316 | 432.16166 | C19 H28 O11 | -3.47 | [M+NH_4_]^+^ | 2-(4-Hydroxyphenyl)ethyl 6-O-[(2R,3R,4R)-3,4-dihydroxy-4-(hydroxymethyl)tetrahydro-2-furanyl]-beta-D-glucopyranoside | 187.0752,147.0439,121.0648,97.0283,85.0284 | Others | Others |
| M76 | 8.50 | 138.03169 | 138.03116 | C7 H6 O3 | -3.84 | [M-H]^-^ | Salicylic acid | 137.0231,93.0335 | Phenols | Phenols |
| M77 | 8.53 | 264.14739 | 264.1478 | C14 H20 N2 O3 | 1.55 | [M+H]^+^ | 2-(2-amino-3-methylbutanamido)-3-phenylpropanoic acid | 120.0809,103.0543,72.0809 | Amino acids and derivatives | Amino acids and derivatives |
| M78 | 8.62 | 176.06847 | 176.06809 | C7 H12 O5 | -2.16 | [M-H]^-^ | 2-Isopropylmalic acid | 157.0494,115.0389,113.0597,85.0649 | Organic acids and derivatives | Organic acids and derivatives |
| M79 | 8.62 | 338.10017 | 338.0985 | C16 H18 O8 | -4.94 | [M-H]^-^ | 1,3,4-Trihydroxy-5-{[(2E)-3-(4-hydroxyphenyl)-2-propenoyl]oxy}cyclohexanecarboxylic acid | 191.0545,163.0387,119.0491 | Organic acids and derivatives | Organic acids and derivatives |
| M80 | 8.67 | 180.04226 | 180.04205 | C9 H8 O4 | -1.17 | [M-H_2_O+H]^+^ | 3-Hydroxy-7-methoxy-2-benzofuran-1(3H)-one | 135.0443,117.0337,89.0387 | Others | Others |
| M81 | 8.71 | 354.09508 | 354.09449 | C16 H18 O9 | -1.67 | [M-H]^-^ | Chlorogenic acid | 191.0549,135.0441,93.0337 | Phenols | Phenols |
| M82 | 8.84 | 834.49769 | 834.49605 | C42 H74 O16 | -1.97 | [M+FA-H]^-^ | 509^#^-(Glc-Glc) | 671.4347,509.3824,221.0638 | Saponins | Others |
| M83 | 8.89 | 980.55559 | 980.55136 | C48 H84 O20 | -4.31 | [M+FA-H]^-^ | 509^#^-Glc-(Glc-Rha) | 979.5421,817.4904,799.4803,671.4337,653.4229,509.3828,205.0705 | Saponins | Others |
| M84 | 8.90 | 368.11073 | 368.10934 | C17 H20 O9 | -3.78 | [M-H]^-^ | (1S,3R,4R,5R)-1,3,4-trihydroxy-5-{[(2E)-3-(4-hydroxy-3-methoxyphenyl)prop-2-enoyl]oxy}cyclohexane-1-carboxylic acid | 193.0494,134.0363,93.0339 | Organic acids and derivatives | Organic acids and derivatives |
| M85 | 9.01 | 980.55559 | 980.55157 | C48 H84 O20 | -4.10 | [M+FA-H]^-^ | 509^#^-Glc-Glc-Rha | 799.4815 | Saponins | Others |
| M86 | 9.03 | 432.16316 | 432.16373 | C19 H28 O11 | 1.32 | [M-H]^-^ | 2-(4-Hydroxyphenyl)ethyl 6-O-[(2R,3R,4R)-3,4-dihydroxy-4-(hydroxymethyl)tetrahydro-2-furanyl]-beta-D-glucopyranoside | 191.0546,149.0446,131.0337,89.0237,71.0132 | Others | Others |
| M87 | 9.05 | 386.19407 | 386.1933 | C19 H30 O8 | -1.99 | [M+NH_4_]^+^ | (4S)-4-hydroxy-3,5,5-trimethyl-4-[(1E)-3-{[(2R,3R,4S,5S,6R)-3,4,5-trihydroxy-6-(hydroxymethyl)oxan-2-yl]oxy}but-1-en-1-yl]cyclohex-2-en-1-one | 207.1383,177.0549,145.0287,123.1169,85.0285 | Others | Others |
| M88 | 9.12 | 450.11621 | 450.11524 | C21 H22 O11 | -2.15 | [M-H]^-^ | 3,5-Dihydroxy-2-(4-hydroxyphenyl)-4-oxo-3,4-dihydro-2H-chromen-7-yl hexopyranoside | 287.0561,259.0596,125.0232 | Others | Others |
| M89 | 9.13 | 552.18429 | 552.18203 | C26 H32 O13 | -4.09 | [M-H]^-^ | Methyl (1S,4aS,5R,7S,7aS)-1-(β-D-glucopyranosyloxy)-7-hydroxy-5-{[(2E)-3-(4-hydroxyphenyl)-2-propenoyl]oxy}-7-methyl-1,4a,5,6,7,7a-hexahydrocyclopenta[c]pyran-4-carboxylate | 341.1007,193.0491,134.0361 | Carbohydrates and derivatives | Carbohydrates and derivatives |
| M90 | 9.15 | 540.22068 | 540.22316 | C26 H36 O12 | 4.59 | [M+NH_4_]^+^ | (2S,3R,4S,5S,6R)-2-[4-[(1S,2R)-1,3-dihydroxy-2-[4-(3-hydroxypropyl)-2-methoxyphenoxy]propyl]-2-methoxyphenoxy]-6-(hydroxymethyl)oxane-3,4,5-triol | 287.1279,189.0912,207.1017,161.0598,137.0599 | Others | Others |
| M91 | 9.19 | 242.12666 | 242.12589 | C11 H18 N2 O4 | -3.18 | [M-H]^-^ | 3-[(2S,5S)-5-[(2S)-butan-2-yl]-3,6-dioxopiperazin-2-yl]propanoic acid | 197.1281,141.1021,112.0393,82.0289 | Amino acids and derivatives | Amino acids and derivatives |
| M92 | 9.20 | 354.09508 | 354.09456 | C16 H18 O9 | -1.47 | [M-H]^-^ | Chlorogenic acid | 191.0549,161.0232,85.0286 | Phenols | Phenols |
| M93 | 9.22 | 382.1839 | 382.18231 | C16 H30 O10 | -4.16 | [M+FA-H]^-^ | 3-Methylbutyl 6-O-Α-α-L-arabinopyranosyl-β-D-glucopyranoside | 381.1744,249.1327,161.0444,101.0233 | Carbohydrates and derivatives | Carbohydrates and derivatives |
| M94 | 9.23 | 180.04226 | 180.04178 | C9 H8 O4 | -2.67 | [M-H]^-^ | Caffeic acid | 135.0440,117.0333,107.0492 | Organic acids and derivatives | Organic acids and derivatives |
| M95 | 9.29 | 328.14231 | 328.14319 | C18 H20 N2 O4 | 2.68 | [M+H]^+^ | N1-(2-{2-[2-(acetylamino)phenoxy]ethoxy}phenyl)acetamide | 136.0759,119.0493,91.0543 | Others | Carboxamides |
| M96 | 9.37 | 382.1839 | 382.18293 | C16 H30 O10 | -2.54 | [M+NH_4_]^+^ | 3-Methylbutyl 6-O-α-L-arabinopyranosyl-β-D-glucopyranoside | 145.0696,127.0391,97.0285,85.0284 | Carbohydrates and derivatives | Carbohydrates and derivatives |
| M97 | 9.39 | 338.10017 | 338.09887 | C16 H18 O8 | -3.85 | [M-H]^-^ | 1,3,4-Trihydroxy-5-{[(2E)-3-(4-hydroxyphenyl)-2-propenoyl]oxy}cyclohexanecarboxylic acid | 191.0546,163.0387,119.0491,93.0336 | Organic acids and derivatives | Organic acids and derivatives |
| M98 | 9.41 | 382.1839 | 382.18274 | C16 H30 O10 | -3.04 | [M+FA-H]^-^ | 3-Methylbutyl 6-O-Α-α-L-arabinopyranosyl-β-D-glucopyranoside | 381.1742,249.1327,159.0286,101.0235 | Carbohydrates and derivatives | Carbohydrates and derivatives |
| M99 | 9.45 | 242.12666 | 242.12553 | C11 H18 N2 O4 | -4.67 | [M-H]^-^ | 3-[(2S,5S)-5-[(2S)-butan-2-yl]-3,6-dioxopiperazin-2-yl]propanoic acid | 241.1178,197.1279,141.1020,112.0394,82.0288 | Amino acids and derivatives | Amino acids and derivatives |
| M100 | 9.50 | 816.48712 | 816.48535 | C42 H72 O15 | -2.17 | [M+FA-H]^-^ | OT-Glc-Glc | 653.4265,635.4101 | Saponins | OT |
| M101 | 9.50 | 948.56577 | 948.56456 | C48 H84 O18 | -1.28 | [M+FA-H]^-^ | 477^#^-Glc-Glc-Rha | 947.5169,785.4586,767.45550,477.3562 | Saponins | Others |
| M102 | 9.58 | 962.54503 | 962.54293 | C48 H82 O19 | -2.18 | [M+FA-H]^-^ | 491^#^-Glc-(Glc-Rha) | 961.5328,799.4791,781.4702,653.4227,635.4132,491.3712,391.2826,205.0705 | Saponins | Others |
| M103 | 9.61 | 540.22068 | 540.22032 | C26 H36 O12 | -0.67 | [M+NH_4_]^+^ | 4-{(1S,2R)-1,3-Dihydroxy-2-[4-(3-hydroxypropyl)-2-methoxyphenoxy]propyl}-2-methoxyphenyl-β-D-glucopyranoside | 287.12827,207.1019,189.0913,163.0758,161.0601 | Others | Others |
| M104 | 9.62 | 194.05791 | 194.05735 | C10 H10 O4 | -2.89 | [M-H_2_O+H]^+^ | 3-(2-Hydroxy-4-methoxy-phenyl)-acrylic acid | 149.0601,145.0287,117.0337,89.0387 | Phenols | Phenols |
| M105 | 9.62 | 368.11073 | 368.11149 | C17 H20 O9 | 2.06 | [M+H]^+^ | (1S,3R,4R,5R)-1,3,4-trihydroxy-5-{[(2E)-3-(4-hydroxy-3-methoxyphenyl)prop-2-enoyl]oxy}cyclohexane-1-carboxylic acid | 177.0550,145.0287,117.0337 | Organic acids and derivatives | Organic acids and derivatives |
| M106 | 9.62 | 122.03678 | 122.03624 | C7 H6 O2 | -4.42 | [M-H]^-^ | 4-Hydroxybenzaldehyde | 121.0283,93.0336,92.0257 | Phenols | Phenols |
| M107 | 9.62 | 428.16825 | 428.17013 | C20 H28 O10 | 4.39 | [M+FA-H]^-^ | (2E)-3-Phenyl-2-propen-1-yl 6-O-beta-D-arabinofuranosyl-beta-D-glucopyranoside | 191.0545,99.0077,89.0234 | Others | Others |
| M108 | 9.63 | 368.11073 | 368.11012 | C17 H20 O9 | -1.66 | [M+Na]^+^ | Cnidioside A | 391.1001,149.0235 | Carbohydrates and derivatives | Carbohydrates and derivatives |
| M109 | 9.73 | 416.16825 | 416.16786 | C19 H28 O10 | -0.94 | [M+NH_4_]^+^ | (2S,3R,4S,5R)-2-{[(2R,3R,4S,5S,6R)-4,5-dihydroxy-6-(hydroxymethyl)-2-(2-phenylethoxy)oxan-3-yl]oxy}oxane-3,4,5-triol | 163.0607,145.0497,127.0392,97.0285,85.0285 | Others | Others |
| M110 | 9.79 | 286.05 | 286.04943 | C11 H9 F3 N4 S | -1.99 | [M+H]^+^ | 4-(methylthio)-6-[4-(trifluoromethyl)phenyl]-1,3,5-triazin-2-amine | 287.0558,153.0185 | Others | Others |
| M111 | 9.79 | 610.15338 | 610.15471 | C27 H30 O16 | 2.18 | [M+H]^+^ | 4-(5,7-Dihydroxy-4-oxo-4H-chromen-2-yl)-2-(β-D-glucopyranosyloxy)phenyl β-D-glucopyranoside | 287.0557,91.0390,85.0286 | Others | Others |
| M112 | 9.83 | 338.10017 | 338.09928 | C16 H18 O8 | -2.63 | [M-H]^-^ | 1,3,4-Trihydroxy-5-{[(2E)-3-(4-hydroxyphenyl)-2-propenoyl]oxy}cyclohexanecarboxylic acid | 191.0550,163.0390,119.0492,93.0337,85.0286,67.0181 | Organic acids and derivatives | Organic acids and derivatives |
| M113 | 9.83 | 173.10519 | 173.10478 | C8 H15 N O3 | -2.37 | [M-H]^-^ | 2-(Acetylamino) hexanoic acid | 130.08626 | Amino acids and derivatives | Amino acids and derivatives |
| M114 | 9.87 | 978.53994 | 978.53788 | C48 H82 O20 | -2.11 | [M+FA-H]^-^ | 507^#^-Glc-(Glc-Rha) | 977.5286,815.4779,797.4655,619.3814,471.3456,391.2823,205.0705 | Saponins | Others |
| M115 | 9.87 | 1124.59785 | 1124.59307 | C54 H92 O24 | -4.25 | [M+FA-H]^-^ | OT-Glc-Glc-Glc-Rha | 1123.5843,977.5288,815.4825 | Saponins | OT |
| M116 | 10.00 | 960.49299 | 960.49726 | C47 H76 O20 | 4.45 | [M+FA-H]^-^ | 489^#^-Glc-Glc-Rha | 779.4548,633.3987,471.3454 | Saponins | Others |
| M117 | 10.02 | 368.11073 | 368.11035 | C17 H20 O9 | -1.03 | [M-H]^-^ | (1S,3R,4R,5R)-1,3,4-trihydroxy-5-{[(2E)-3-(4-hydroxy-3-methoxyphenyl)prop-2-enoyl]oxy}cyclohexane-1-carboxylic acid | 191.0547,173.0442,134.0362,93.0337 | Organic acids and derivatives | Organic acids and derivatives |
| M118 | 10.03 | 948.52938 | 948.52758 | C47 H80 O19 | -1.90 | [M+FA-H]^-^ | OT-Glc-(Glc-Xyl) | 947.5186,815.4765,797.4679,653.4241,635.4129,491.3717,191.0547 | Saponins | OT |
| M119 | 10.15 | 522.21011 | 522.21144 | C26 H34 O11 | 2.55 | [M+FA-H]^-^ | Lariciresinol 4-O-glucoside | 359.1483,329.1373,175.0752,160.0518 | Carbohydrates and derivatives | Carbohydrates and derivatives |
| M120 | 10.23 | 816.48712 | 816.48634 | C42 H72 O15 | -0.96 | [M+FA-H]^-^ | 491^#^-20-Glc-6-Glc | 815.4738,653.4237 | Saponins | Others |
| M121 | 10.28 | 962.54503 | 962.54302 | C48 H82 O19 | -2.09 | [M+FA-H]^-^ | 491^#^-20-Glc-3-(Glc-Rha) | 961.5323,815.4759,799.4792,653.4237,635.4152,491.3712,205.0705 | Saponins | Others |
| M122 | 10.30 | 804.48712 | 804.48574 | C41 H72 O15 | -1.72 | [M+FA-H]^-^ | 509^#^-(Glc-Xyl) | 803.4755,671.4340,653.4226,509.3819,391.2832,191.0546 | Saponins | Others |
| M123 | 10.32 | 672.44486 | 672.44574 | C36 H64 O11 | 1.31 | [M+FA-H]^-^ | 509^#^-Glc | 671.4392,509.3859,391.2860 | Saponins | Others |
| M124 | 10.36 | 818.50277 | 818.50206 | C42 H74 O15 | -0.87 | [M+FA-H]^-^ | 509^#^-(Glc-Rha) | 817.4898,671.4334,653.4226,509.3813,391.2829,205.0701 | Saponins | Others |
| M125 | 10.40 | 207.08954 | 207.08886 | C11 H13 N O3 | -3.28 | [M-H]^-^ | afalanine | 164.0703,147.0438,91.0544,70.0290,58.0290 | Amino acids and derivatives | Amino acids and derivatives |
| M126 | 10.52 | 524.22576 | 524.22703 | C26 H36 O11 | 2.42 | [M+FA-H]^-^ | 3-(4-{[1,3-Dihydroxy-1-(4-hydroxy-3-methoxyphenyl)-2-propanyl]oxy}-3-methoxyphenyl)propyl 6-deoxy-alpha-L-mannopyranoside | 523.2187,361.1635,165.0542,101.0233 | Others | Others |
| M127 | 10.52 | 368.11073 | 368.10977 | C17 H20 O9 | -2.61 | [M-H_2_O-H]^-^ | 1,3,5-trihydroxy-4-{[(2E)-3-(3-hydroxy-4-methoxyphenyl)prop-2-enoyl]oxy}cyclohexane-1-carboxylic acid | 193.0491,178.0254,134.0361,93.0336 | Organic acids and derivatives | Organic acids and derivatives |
| M128 | 10.54 | 520.19446 | 520.19381 | C26 H32 O11 | -1.25 | [M+FA-H]^-^ | 4-[4-(4-Hydroxy-3-methoxyphenyl)tetrahydro-1H,3H-furo[3,4-c]furan-1-yl]-2-methoxyphenyl hexopyranoside | 357.1321,151.0388,136.0153 | Others | Others |
| M129 | 10.60 | 246.10044 | 246.09962 | C13 H14 N2 O3 | -3.33 | [M-H]^-^ | 2-(acetylamino)-3-(1H-indol-3-yl) propanoic acid | 203.0811,116.0499,74.0239 | Amino acids and derivatives | Amino acids and derivatives |
| M130 | 10.74 | 902.48752 | 902.48345 | C45 H74 O18 | -4.51 | [M+FA-H]^-^ | OT-6-Glc-Glc-Mal | 797.4607,653.4254,635.4155, | Saponins | OT |
| M131 | 10.77 | 1108.60294 | 1108.59925 | C54 H92 O23 | -3.33 | [M+FA-H]^-^ | PPT-(Glc-Glc)-Glc-Rha | 1107.5878,961.5322,945.5368,799.4798,637.4287,475.3760,221.0638 | Saponins | PPT |
| M132 | 10.81 | 214.12051 | 214.12087 | C11 H18 O4 | 1.68 | [M+H]^+^ | (2S)-2-Hexyl-3-methylenesuccinic acid | 119.0856,97.0649,81.0699 | Organic acids and derivatives | Organic acids and derivatives |
| M133 | 10.83 | 962.54503 | 962.54342 | C48 H82 O19 | -1.67 | [M+FA-H]^-^ | PPT-(Glc-Glc)-Glc | 961.5320,799.4765,781.4769,221.0651 | Saponins | PPT |
| M134 | 10.84 | 522.21011 | 522.21164 | C26 H34 O11 | 2.93 | [M+NH_4_]^+^ | 2-(hydroxymethyl)-6-{5-[3-(hydroxymethyl)-5-(3-hydroxypropyl)-7-methoxy-2,3-dihydro-1-benzofuran-2-yl]-2-methoxyphenoxy}oxane-3,4,5-triol | 313.1441,177.0912,163.0756,137.0601 | Others | Others |
| M135 | 10.87 | 368.11073 | 368.10955 | C17 H20 O9 | -3.21 | [M-H_2_O-H]^-^ | 1,3,5-trihydroxy-4-{[(2E)-3-(3-hydroxy-4-methoxyphenyl)prop-2-enoyl]oxy}cyclohexane-1-carboxylic acid | 193.0491,134.0360,93.0336 | Organic acids and derivatives | Organic acids and derivatives |
| M136 | 10.92 | 1108.60294 | 1108.59924 | C54 H92 O23 | -3.34 | [M+FA-H]^-^ | PPT-(Glc-Glc)-Glc-Rha | 1107.5869,961.5312,783.4853,637.4286,475.3768,391.2835,221.0653 | Saponins | PPT |
| M137 | 11.12 | 932.53447 | 932.53329 | C47 H80 O18 | -1.27 | [M+FA-H]^-^ | PPT-20-(Glc-Xyl)-6-Glc | 931.5222,799.4816,637.4292,475.3764,391.2825,191.0547 | Saponins | PPT |
| M138 | 11.14 | 1078.59237 | 1078.58958 | C53 H90 O22 | -2.59 | [M+FA-H]^-^ | PPT-(Glc-Rha)-(Glc-Xyl) | 1077.5776,945.5354,783.4821,637.4303,475.3788,191.0543 | Saponins | PPT |
| M139 | 11.15 | 480.15801 | 480.15723 | C28 H24 Cl F3 N2 | -1.62 | [M+H]^+^ | 4-(4-benzhydrylpiperidino)-8-chloro-2-(trifluoromethyl)quinoline | 481.1690,317.0856 | Others | Others |
| M140 | 11.26 | 962.54503 | 962.54298 | C48 H82 O19 | -2.13 | [M+FA-H]^-^ | PPT-(Glc-Glc)-Glc | 961.5316,799.4802,637.4282,475.3760,221.0655 | Saponins | PPT |
| M141 | 11.35 | 932.53447 | 932.53406 | C47 H80 O18 | -0.44 | [M+FA-H]^-^ | PPT-20-(Glc-Xyl)-6-Glc | 931.5215,799.4799,637.4286,475.3764,191.0545 | Saponins | PPT |
| M142 | 11.36 | 1108.60294 | 1108.60083 | C54 H92 O23 | -1.90 | [M+FA-H]^-^ | PPT-(Glc-Glc)-Glc-Rha | 1107.5881,961.5332,945.5352,783.4890,637.4306,475.3764,221.0650 | Saponins | PPT |
| M143 | 11.38 | 1142.60842 | 1142.60508 | C54 H94 O25 | -2.92 | [M+FA-H]^-^ | 493^#^-(Glc-Glc)-Glc-Glc | 1141.5943,979.5488,817.4897,799.4813,655.4384,493.3881,221.0649 | Saponins | Others |
| M144 | 11.41 | 145.05276 | 145.05216 | C9 H7 N O | -4.14 | [M-H]^-^ | 4-Indolecarbaldehyde | 144.0462,102.1629 | Others | Indoles |
| M145 | 11.41 | 1078.59237 | 1078.58931 | C53 H90 O22 | -2.84 | [M+FA-H]^-^ | PPT-Glc-Rha-(Glc-Xyl) | 1077.5783,945.5361,931.5186,783.4894,637.4298,191.0549 | Saponins | PPT |
| M146 | 11.55 | 932.53447 | 932.53307 | C47 H80 O18 | -1.50 | [M+FA-H]^-^ | PPT-Glc-(Glc-Xyl) | 931.5217,799.4807,637.4289,475.3767,391.2832,191.0544 | Saponins | PPT |
| M147 | 11.61 | 962.54503 | 962.5432 | C48 H82 O19 | -1.90 | [M+FA-H]^-^ | PPT-Glc-Glc-Glc | 961.5317,799.4810,637.4278,475.3762 | Saponins | PPT |
| M148 | 11.61 | 188.10486 | 188.10397 | C9 H16 O4 | -4.73 | [M-H]^-^ | Azelaic acid | 187.0981,143.1065,125.0960,97.0649 | Lipids | Fatty acids and derivatives |
| M149 | 11.68 | 800.49221 | 800.49213 | C42 H72 O14 | -0.10 | [M+FA-H]^-^ | 491^#^-(Glc-Rha) | 799.4811,653.4246,491.3716,391.2833,205.0701 | Saponins | Others |
| M150 | 11.82 | 1108.60294 | 1108.60047 | C54 H92 O23 | -2.23 | [M+FA-H]^-^ | PPT-Glc-Glc-Glc-Rha | 1107.5882,961.5307,945.5385,783.4884,637.4301,475.3770 | Saponins | PPT |
| M151 | 11.84 | 1122.5822 | 1122.57913 | C54 H90 O24 | -2.73 | [M+FA-H]^-^ | 473^#^-(Glc-Glc)-Glc-Glc | 1121.5665,959.5163,797.4640,631.4935,473.3606,221.0650 | Saponins | Others |
| M152 | 11.85 | 962.54503 | 962.54322 | C48 H82 O19 | -1.88 | [M+FA-H]^-^ | PPT-Glc-Glc-Glc | 961.5322,799.4837,637.4287 | Saponins | PPT |
| M153 | 11.86 | 1228.60881 | 1228.60325 | C57 H96 O28 | -4.53 | [M-H]^-^ | 493^#^-(Glc-Glc)-Glc-Glc-Mal | 1141.5938,1123.5828,817.4924,799.4822,221.0652 | Saponins | Others |
| M154 | 11.91 | 1142.60842 | 1142.61194 | C54 H94 O25 | 3.08 | [M+NH_4_]^+^ | 493^#^-(Glc-Glc)-Glc-Glc | 441.3736,423.3631 | Saponins | Others |
| M155 | 11.93 | 1124.59785 | 1124.59371 | C54 H92 O24 | -3.68 | [M+FA-H]^-^ | PPT-(Glc-Glc)-Glc-Glc | 1123.5833,961.5306,799.4829,221.0648 | Saponins | PPT |
| M156 | 12.12 | 138.03169 | 138.03123 | C7 H6 O3 | -3.33 | [M-H]^-^ | Salicylic acid | 1137.0259,93.0335,65.0388 | Phenols | Phenols |
| M157 | 12.19 | 1014.57633 | 1014.57782 | C52 H86 O19 | 1.47 | [M+FA-H]^-^ | PPT-Glc-Glc-Rha-Butenoyl | 945.5362 | Saponins | PPT |
| M158 | 12.20 | 800.49221 | 800.49386 | C42 H72 O14 | 2.06 | [M+FA-H]^-^ | Ginsenoside Rg_1_* | 799.4807,637.4291,475.3770,391.2838, | Saponins | PPT |
| M159 | 12.21 | 946.55012 | 946.55062 | C48 H82 O18 | 0.53 | [M+FA-H]^-^ | Ginsenoside Re* | 945.5378,799.4813,7834865,637.4296,475.3775,391.2839,205.0708 | Saponins | PPT |
| M160 | 12.51 | 1228.60881 | 1228.60644 | C57 H96 O28 | -1.93 | [M-H]^-^ | 493^#^-(Glc-Glc)-Glc-Glc-Mal | 1141.5938,1123.5850,817.4894,799.4801,655.4429,493.3862,375.6520,221.0652 | Saponins | Others |
| M161 | 12.86 | 786.47656 | 786.47624 | C41 H70 O14 | -0.41 | [M+FA-H]^-^ | OT-(Glc-Xyl) | 785.4665,653.4250,191.0551 | Saponins | OT |
| M162 | 12.95 | 980.55559 | 980.55348 | C48 H84 O20 | -2.15 | [M+FA-H]^-^ | 493^#^-Glc-Glc-Glc | 979.5418,817.4907,799.4804,655.4392,637.4292,493.3885 | Saponins | Others |
| M163 | 12.97 | 1124.59785 | 1124.5941 | C54 H92 O24 | -3.33 | [M+FA-H]^-^ | PPT-(Glc-Glc)-Glc-Glc | 1123.5816,961.5301,799.4806,637.4298,475.3758,221.0649 | Saponins | PPT |
| M164 | 12.99 | 448.23085 | 448.23143 | C21 H36 O10 | 1.29 | [M+NH_4_]^+^ | (2R,3S,4S,5R,6R)-2-({[(2R,3R,4R)-3,4-dihydroxy-4-(hydroxymethyl)oxolan-2-yl]oxy}methyl)-6-{[(2E)-3,7-dimethylocta-2,6-dien-1-yl]oxy}oxane-3,4,5-triol | 137.3277,115.0391,81.0699 | Others | Others |
| M165 | 13.04 | 448.23085 | 448.22977 | C21 H36 O10 | -2.41 | [M+FA-H]^-^ | 2-[3,8-Dihydroxy-8-(hydroxymethyl)-3-methyl-2-oxodecahydro-5-azulenyl]-2-propanyl hexopyranoside | 447.2239,293.0846,161.0441 | Carbohydrates and derivatives | Carbohydrates and derivatives |
| M166 | 13.08 | 886.4926 | 886.4895 | C45 H74 O17 | -3.50 | [M+FA-H]^-^ | PPT-20-Glc-6-Glc-Mal | 799.4803,781.4694,637.4285,619.4182,475.3767,391.2828 | Saponins | PPT |
| M167 | 13.08 | 842.50277 | 842.50135 | C44 H74 O15 | -1.69 | [M+FA-H]^-^ | PPT-Glc-Glc-Ac | 637.4294,619.4191,475.3770 | Saponins | PPT |
| M168 | 13.19 | 800.49221 | 800.48885 | C42 H72 O14 | -4.20 | [M+Cl]^-^ | PPT+Glc-Glc | 799.4804 | Saponins | PPT |
| M169 | 13.19 | 800.49221 | 800.49264 | C42 H72 O14 | 0.54 | [M+FA-H]^-^ | OT-6-(Glc-Rha) | 799.4795,653.4229,491.3718,205.0704 | Saponins | OT |
| M170 | 13.22 | 1032.55051 | 1032.54781 | C51 H84 O21 | -2.61 | [M+FA-H]^-^ | PPT-20-Glc-3-(Glc-Rha)-Mal | 945.5359,927.5257,799.4791,783.4848,637.4278,619.4173,475.3761,391.2826 | Saponins | PPT |
| M171 | 13.54 | 1124.59785 | 1124.5945 | C54 H92 O24 | -2.98 | [M+FA-H]^-^ | PPT-(Glc-Glc)-Glc-Glc | 1123.5835,781.4701,221.0650 | Saponins | PPT |
| M172 | 13.71 | 1094.58729 | 1094.58385 | C53 H90 O23 | -3.14 | [M+FA-H]^-^ | PPT-(Glc-Glc)-(Glc-Xyl) | 1093.5737,961.5305,799.482,637.4306,475.3768,191.0547 | Saponins | PPT |
| M173 | 13.80 | 1032.55051 | 1032.54782 | C51 H84 O21 | -2.61 | [M-H]^-^ | PPT-Glc-(Glc-Rha)-Mal | 945.5373,927.5269,783.4856,637.4287,619.4183,475.3768,391.2835,205.0701 | Saponins | PPT |
| M174 | 13.87 | 916.53955 | 916.53788 | C47 H80 O17 | -1.82 | [M+FA-H]^-^ | PPT-Glc-Rha-Xyl | 915.5283,753.4749,607.4154,475.3771 | Saponins | PPT |
| M175 | 13.92 | 962.54503 | 962.54288 | C48 H82 O19 | -2.23 | [M+FA-H]^-^ | OT-Glc-Glc-Rha | 961.5316,815.4752,799.4818,653.4227 | Saponins | OT |
| M176 | 13.94 | 770.48164 | 770.48097 | C41 H70 O13 | -0.87 | [M+FA-H]^-^ | PPT-Glc-Xyl | 607.4231,475.3762 | Saponins | PPT |
| M177 | 13.95 | 886.4926 | 886.48939 | C45 H74 O17 | -3.62 | [M-H]^-^ | PPT-20-Glc-6-Glc-Mal | 799.4797,781.4701,637.4293,619.4183,475.3766,391.2829 | Saponins | PPT |
| M178 | 14.20 | 1124.59785 | 1124.59422 | C54 H92 O24 | -3.23 | [M+FA-H]^-^ | PPT-(Glc-Glc)-Glc-Glc | 1123.5832,961.5333,799.4799,221.0651 | Saponins | PPT |
| M179 | 14.23 | 1210.59825 | 1210.59261 | C57 H94 O27 | -4.66 | [M-H]^-^ | PPT-(Glc-Glc)-Glc-Glc-Mal | 1123.5804,781.4694,637.4368,221.0654 | Saponins | PPT |
| M180 | 14.43 | 1124.59785 | 1124.59364 | C54 H92 O24 | -3.74 | [M+FA-H]^-^ | PPT-(Glc-Glc)-Glc-Glc | 1123.5844,961.5302,799.4824,781.4695,221.0652 | Saponins | PPT |
| M181 | 14.53 | 770.48164 | 770.48168 | C41 H70 O13 | 0.05 | [M+FA-H]^-^ | PPT-Glc-Xyl | 637.4307 | Saponins | PPT |
| M182 | 14.59 | 1140.59277 | 1140.58908 | C54 H92 O25 | -3.24 | [M+FA-H]^-^ | OT-(Glc-Glc)-Glc-Glc | 797.4653,765.4382,221.0652 | Saponins | OT |
| M183 | 14.71 | 1094.58729 | 1094.58284 | C53 H90 O23 | -4.07 | [M+FA-H]^-^ | PPT-Glc-Glc-(Glc-Xyl)-Mal | 1093.5723,931.5233,799.4838,781.4707,191.0551 | Saponins | PPT |
| M184 | 14.76 | 916.53955 | 916.53783 | C47 H80 O17 | -1.88 | [M+FA-H]^-^ | PPT-(Glc-Rha)-Xyl | 915.5270,783.4853,637.4284,619.4179,475.3767,391.2831,205.0702 | Saponins | PPT |
| M185 | 14.82 | 1122.5822 | 1122.57814 | C54 H90 O24 | -3.62 | [M+FA-H]^-^ | 473^#^-(Glc-Glc)-Glc-Glc | 1121.5679,959.5164,843.5287,797.4671,473.3590,221.0652 | Saponins | Others |
| M186 | 14.85 | 800.49221 | 800.49162 | C42 H72 O14 | -0.74 | [M+FA-H]^-^ | OT-(Glc-Rha) | 799.4801,653.4230,635.4117,491.3712,205.0713 | Saponins | OT |
| M187 | 14.89 | 770.48164 | 770.48158 | C41 H70 O13 | -0.08 | [M+FA-H]^-^ | PPT-Glc-Xyl | 637.4286,475.3767,391.2834 | Saponins | PPT |
| M188 | 15.01 | 842.50277 | 842.50213 | C44 H74 O15 | -0.76 | [M+FA-H]^-^ | PPT-20-Glc-6-Glc-Ac | 799.4787,781.4689,637.4279,619.4176,475.3764,391.2829 | Saponins | PPT |
| M189 | 15.32 | 988.56068 | 988.55845 | C50 H84 O19 | -2.26 | [M+FA-H]^-^ | PPT-20-Glc-6-(Glc-Rha)-Ac | 945.5364,927.5261,799.4805,783.4854,637.4282,475.3764,391.2831,205.0701 | Saponins | PPT |
| M190 | 15.34 | 944.53447 | 944.53212 | C48 H80 O18 | -2.49 | [M+FA-H]^-^ | 473^#^-Glc-Glc-Rha | 943.5220,797.4678,635.4130,617.4054 | Saponins | Others |
| M191 | 15.55 | 962.54503 | 962.54282 | C48 H82 O19 | -2.30 | [M+FA-H]^-^ | PPT-Glc-Glc-Glc | 961.5335,799.4830,781.4699 | Saponins | PPT |
| M192 | 15.78 | 816.48712 | 816.48638 | C42 H72 O15 | -0.91 | [M+FA-H]^-^ | OT-6-(Glc-Glc) | 815.4781,653.4231,491.3711,221.0651 | Saponins | OT |
| M193 | 16.00 | 962.54503 | 962.54283 | C48 H82 O19 | -2.29 | [M+FA-H]^-^ | PPT-20-Glc-6-(Glc-Glc) | 961.5322,799.4805,637.4284,475.3760,391.2837,221.0651 | Saponins | PPT |
| M194 | 16.13 | 1254.62446 | 1254.62548 | C59 H98 O28 | 0.81 | [M+FA-H]^-^ | 473^#^-(Glc-Glc)-Glc-(Glc-Xyl) | 1253.6461,1091.5943,929.5451,767.4959,605.4398 | Saponins | Others |
| M195 | 16.47 | 990.5399 | 990.54368 | C49 H82 O20 | 3.82 | [M+NH_4_-H_2_O]^+^ | PPD-3-Glc-Glc-Glc-Formyl | 990.5707,972.5799,548.2091,384.1771,342.1661 | Saponins | PPD |
| M196 | 16.77 | 930.5552 | 930.55355 | C48 H82 O17 | -1.77 | [M+FA-H]^-^ | PPT-(Glc-Rha)-Rha | 929.5397,783.4810,637.4281,475.3782,205.0705 | Saponins | PPT |
| M197 | 16.86 | 962.54503 | 962.54262 | C48 H82 O19 | -2.50 | [M+FA-H]^-^ | 491^#^-Glc-Glc-Rha | 961.5326,799.4801,781.4686, | Saponins | Others |
| M198 | 16.90 | 974.54503 | 974.54163 | C49 H82 O19 | -3.49 | [M+FA-H]^-^ | 457^#^-Glc-Glc-Rha | 927.5258,781.4686,619.4186 | Saponins | Others |
| M199 | 16.94 | 818.50277 | 818.50173 | C42 H74 O15 | -1.27 | [M+FA-H]^-^ | 493^#^-Glc-Glc | 655.4382 | Saponins | Others |
| M200 | 17.02 | 842.50277 | 842.50172 | C44 H74 O15 | -1.25 | [M+FA-H]^-^ | PPT-Glc-Glc-Ac | 799.4804,637.4279,619.4190, | Saponins | PPT |
| M201 | 17.02 | 990.5399 | 990.54368 | C49 H82 O20 | 3.82 | [M+NH_4_-H_2_O]^+^ | PPD-3-Glc-Glc-Glc-Formyl | 990.5946,972.5835,548.2094,384.1772,342.1665 | Saponins | PPD |
| M202 | 17.15 | 786.47656 | 786.47593 | C41 H70 O14 | -0.80 | [M+FA-H]^-^ | OT-Glc-Xyl | 785.4649,653.4240,491.3720 | Saponins | OT |
| M203 | 17.46 | 786.47656 | 786.47378 | C41 H70 O14 | -3.53 | [M+FA-H]^-^ | OT-6-(Glc-Xyl) | 785.4642,653.4232,491.3715,191.0549 | Saponins | OT |
| M204 | 17.66 | 1118.54822 | 1118.54288 | C54 H86 O24 | -4.77 | [M-H]^-^ | OA-3-GluA-Glc-28-(Glc-Glc) | 1117.5354,793.4330,569.3815,455.3504 | Saponins | OA |
| M205 | 17.76 | 1250.59316 | 1250.5893 | C59 H94 O28 | -3.09 | [M-H]^-^ | OA-GluA-Glc-Glc-Glc-Xyl | 1249.5788,1099.5412,731.4342,569.3826,455.3503 | Saponins | OA |
| M206 | 17.82 | 800.49221 | 800.48981 | C42 H72 O14 | -3.00 | [M+FA-H]^-^ | Pseudo-ginsenoside F_11_* | 799.4801,653.4235,491.3718,205.7068 | Saponins | OT |
| M207 | 18.05 | 1250.59316 | 1250.5893 | C59 H94 O28 | -3.09 | [M-H]^-^ | OA-GluA-Glc-Glc-Glc-Xyl | 1249.5782,925.4743,731.4397,455.3499 | Saponins | OA |
| M208 | 18.08 | 654.4343 | 654.43532 | C36 H62 O10 | 1.56 | [M+FA-H]^-^ | Pseudo-ginsenoside RT_5_* | 653.4288 | Saponins | OT |
| M209 | 18.18 | 1270.65576 | 1270.65104 | C60 H102 O28 | -3.71 | [M+FA-H]^-^ | PPD-(Glc-Glc)-Glc-Glc-Glc | 1269.6402,1077.5877,945.5370,783.4862,621.4330,221.0648 | Saponins | PPD |
| M210 | 18.52 | 1356.65616 | 1356.64947 | C63 H104 O31 | -4.93 | [M-H]^-^ | PPD-(Glc-Glc)-Glc-Glc-Glc-Mal | 1311.6484,1269.6405,1251.6308,1107.5849,1089.5776,945.5378,221.0654 | Saponins | PPD |
| M211 | 18.62 | 1240.6452 | 1240.64058 | C59 H100 O27 | -3.72 | [M+FA-H]^-^ | PPD-(Glc-Glc)-Glc-(Glc-Xyl) | 1239.6309,1107.5874,221.0647,191.0554 | Saponins | PPD |
| M212 | 18.84 | 1356.65616 | 1356.64947 | C63 H104 O31 | -4.93 | [M-H]^-^ | PPD-(Glc-Glc)-Glc-Glc-Glc-Mal | 1269.6401,1251.6303,1107.5861,1089.5760,783.4851,221.0652 | Saponins | PPD |
| M213 | 18.88 | 770.48164 | 770.48159 | C41 H70 O13 | -0.06 | [M+FA-H]^-^ | PPT-(Glc-Xyl) | 769.4710,637.4296,475.3771,391.2840,191.0552 | Saponins | PPT |
| M214 | 19.03 | 800.49221 | 800.4915 | C42 H72 O14 | -0.89 | [M+FA-H]^-^ | PPT-Glc-Glc | 799.4801,637.4257,475.3779 | Saponins | PPT |
| M215 | 19.30 | 1356.65616 | 1356.64995 | C63 H104 O31 | -4.58 | [M-H]^-^ | PPD-(Glc-Glc)-Glc-Glc-Glc-Mal | 1311.6496,1269.6407,1251.6320,1107.5907,945.5291,783.4832 | Saponins | PPD |
| M216 | 19.31 | 1088.54034 | 1088.53695 | C53 H84 O23 | -3.11 | [M-H]^-^ | OA-GluA-Glc-Glc-Xyl | 1087.5249,937.4746,569.3811,455.3506 | Saponins | OA |
| M217 | 19.35 | 1270.65576 | 1270.64975 | C60 H102 O28 | -4.73 | [M+FA-H]^-^ | PPD-(Glc-Glc)-Glc-Glc-Glc | 1269.6395,1107.5877,945.5365,783.4862,621.4334,221.0648 | Saponins | PPD |
| M218 | 19.58 | 886.4926 | 886.49012 | C45 H74 O17 | -2.80 | [M+FA-H]^-^ | OT-(Glc-Rha)-Mal | 841.4882,799.4810,653.4229,491.3716,205.0706 | Saponins | OT |
| M219 | 19.80 | 770.48164 | 770.4816 | C41 H70 O13 | -0.05 | [M+FA-H]^-^ | PPT-6-(Glc-Xyl) | 637.4283,475.3775,191.0547 | Saponins | PPT |
| M220 | 19.86 | 1092.60802 | 1092.60474 | C54 H92 O22 | -3.00 | [M+FA-H]^-^ | 443^#^-(Glc-Glc)-Glc-Glc | 1091.5944,929.5437,767.4915,605.4397,221.0656 | Saponins | Others |
| M221 | 19.87 | 944.53447 | 944.53235 | C48 H80 O18 | -2.24 | [M+FA-H]^-^ | 457^#^-Glc-Glc-Glc | 943.5226,781.4691,619.4170 | Saponins | Others |
| M222 | 19.97 | 1106.58729 | 1106.58387 | C54 H90 O23 | -3.09 | [M+FA-H]^-^ | 457^#^-20-(Glc-Glc)-3-Glc-Glc | 1105.5731,943.5217,781.4702,619.4781,457.3659,221.0654 | Saponins | Others |
| M223 | 20.02 | 1356.65616 | 1356.64986 | C63 H104 O31 | -4.64 | [M-H]^-^ | PPD-(Glc-Glc)-Glc-Glc-Glc-Mal | 1269.6401,1251.6292,1107.5875,1089.5780,945.5349,783.4827,621.4356,221.0649 | Saponins | PPD |
| M224 | 20.07 | 784.49729 | 784.49351 | C42 H72 O13 | -4.82 | [M+FA-H]^-^ | 20(S)-Ginsenoside Rg_2_* | 783.4854,637.4284,619.4176,475.3766,391.2831,205.0705 | Saponins | PPT |
| M225 | 20.15 | 330.24062 | 330.24134 | C18 H34 O5 | 2.18 | [M+NH4]^+^ | (9Z)-5,8,11-Trihydroxy-9-octadecenoic acid | 155.1069,109.1013,67.0543 | Lipids | Fatty acids and derivatives |
| M226 | 20.17 | 1270.65576 | 1270.65147 | C60 H102 O28 | -3.38 | [M+FA-H]^-^ | PPD-(Glc-Glc)-Glc-Glc-Glc | 1269.6409,1107.5886,945.5356,783.4871,221.0645 | Saponins | PPD |
| M227 | 20.18 | 330.24062 | 330.23946 | C18 H34 O5 | -3.51 | [M-H]^-^ | (15Z)-9,12,13-Trihydroxy-15-octadecenoic acid | 329.2334,229.1429,171.1013,127.1117 | Lipids | Fatty acids and derivatives |
| M228 | 20.23 | 638.43938 | 638.44054 | C36 H62 O9 | 1.82 | [M+FA-H]^-^ | PPT-Glc | 637.4284,475.3764 | Saponins | PPT |
| M229 | 20.31 | 956.49808 | 956.49634 | C48 H76 O19 | -1.82 | [M-H]^-^ | OA-GluA-Glc-Glc | 955.4843,793.4328,731.4334,569.3808,455.3504 | Saponins | OA |
| M230 | 20.40 | 740.43469 | 740.43125 | C39 H64 O13 | -4.65 | [M+Na]^+^ | OT-6-Glc-Mal | 763.4252,719.4359,497.3580,289.0538 | Saponins | OT |
| M231 | 20.47 | 784.49729 | 784.49763 | C42 H72 O13 | 0.43 | [M+FA-H]^-^ | 20(R)-Ginsenoside Rg_2_* | 783.4859,637.4278,619.4188,475.3768,391.2831 | Saponins | PPT |
| M232 | 20.59 | 1194.60333 | 1194.59839 | C57 H94 O26 | -4.14 | [M-H]^-^ | PPD-(Glc-Glc)-Glc-Glc-Mal | 1107.5882,1089.5769,945.5383,783.4854,621.4343,221.0652 | Saponins | PPD |
| M233 | 20.77 | 288.08452 | 288.08534 | C12 H16 O8 | 2.85 | [M+H]^+^ | Maltol-Glucoside | 127.0394,97.0285,85.0285 | Carbohydrates and derivatives | Carbohydrates and derivatives |
| M234 | 20.79 | 1108.60294 | 1108.60008 | C54 H92 O23 | -2.58 | [M+FA-H]^-^ | Ginsenoside Rb_1_* | 1107.5971,945.5371,783.4859,621.4342,459.3823,221.0654 | Saponins | PPD |
| M235 | 20.80 | 1176.62915 | 1176.62341 | C58 H96 O24 | -4.88 | [M+FA-H]^-^ | PPD-Glc-Glc-Glc-Glc-Butenoyl | 1107.5889,945.5374,783.4859 | Saponins | PPD |
| M236 | 21.10 | 956.49808 | 956.49946 | C48 H76 O19 | 1.44 | [M-H]^-^ | OA-GluA-Glc-Glc | 955.4848,793.4339,569.3818 | Saponins | OA |
| M237 | 21.34 | 1216.58528 | 1216.5819 | C57 H93 O26 | -2.78 | [M+Na]^+^ | PPD-20-Glc-Glc-3-Glc-Glc-Mal | 875.4790,831.4893,789.4766,451.1080,407.1168,365.1062 | Saponins | PPD |
| M238 | 21.38 | 1326.64559 | 1326.63897 | C62 H102 O30 | -4.99 | [M-H]^-^ | PPD-(Glc-Glc)-Glc-Glc-Xyl-Mal | 1239.6345,1221.6197,1107.5841,945.5434,783.4861,221.0654 | Saponins | PPD |
| M239 | 21.42 | 1262.62955 | 1262.62417 | C61 H98 O27 | -4.26 | [M-H]^-^ | PPD-(Glc-Glc)-Glc-Glc-Butenoyl-Mal | 1149.5987,1107.5878,1089.5802,945.5374,783.4856,621.4357,221.0649 | Saponins | PPD |
| M240 | 21.50 | 1150.6135 | 1150.61408 | C56 H94 O24 | 0.50 | [M-H]^-^ | PPD-(Glc-Glc)-Glc-Glc-Ac | 1107.5872,1089.5769,945.5368,783.4851,621.4333,459.3815,221.0649 | Saponins | PPD |
| M241 | 21.53 | 1194.60333 | 1194.5975 | C57 H94 O26 | -4.88 | [M+Na]^+^ | PPD-20-Glc-Glc-3-Glc-Glc-Mal | 875.4790,831.4893,789.4766,451.1080,407.1168,365.1062 | Saponins | PPD |
| M242 | 21.68 | 1108.60294 | 1108.59871 | C54 H92 O23 | -3.82 | [M+FA-H]^-^ | PPD-(Glc-Glc)-Glc-Glc | 1107.5888,945.5360,783.4868,221.0649 | Saponins | PPD |
| M243 | 21.79 | 1078.59237 | 1078.58738 | C53 H90 O22 | -4.63 | [M+FA-H]^-^ | Ginsenoside Rc* | 1077.5775,945.5371,783.4857,621.4341,459.3821,221.0654,191.0549 | Saponins | PPD |
| M244 | 21.80 | 794.44526 | 794.44434 | C42 H66 O14 | -1.16 | [M+NH_4_]^+^ | OA-GluA-Glc | 439.3583,393.3524 | Saponins | OA |
| M245 | 21.85 | 1088.54034 | 1088.53667 | C53 H84 O23 | -3.37 | [M-H]^-^ | OA-GluA-Glc-Glc-Xyl | 1087.5253,955.4866,731.4335,569.3813,455.3506 | Saponins | OA |
| M246 | 21.88 | 956.49808 | 956.49911 | C48 H76 O19 | 1.08 | [M-H]^-^ | Ginsenoside Ro***** | 955.4846,793.4326,569.3808,455.3499 | Saponins | OA |
| M247 | 21.94 | 1024.52429 | 1024.51922 | C52 H80 O20 | -4.95 | [M+FA-H]^-^ | OA-GluA-Glc-Glc-Butenoyl | 955.4857,793.4340,569.3819,455.3486 | Saponins | OA |
| M248 | 21.94 | 1194.60333 | 1194.59992 | C57 H94 O26 | -2.85 | [M-H]^-^ | PPD-(Glc-Glc)-Glc-Glc-Mal | 1107.5866,1089.5769,945.5364,783.4854,621.4340,459.3821,221.0653 | Saponins | PPD |
| M249 | 22.15 | 1194.60333 | 1194.5979 | C57 H94 O26 | -4.55 | [M-H]^-^ | PPD-20-(Glc-Glc)-3-Glc-Glc-Mal | 1107.5872,1089.5772,945.5369,783.4855,621.4340,459.3822,221.0654 | Saponins | PPD |
| M250 | 22.23 | 1088.54034 | 1088.53687 | C53 H84 O23 | -3.19 | [M-H]^-^ | OA-3-GluA-Glc-Xyl-28-Glc | 1087.5245,925.4739,731.4334,569.3814,455.3502 | Saponins | OA |
| M251 | 22.31 | 1280.60373 | 1280.59805 | C60 H96 O29 | -4.44 | [M-H]^-^ | PPD-(Glc-Glc)-Glc-Glc-2Mal | 1149.5983,1107.5880,1089.5777,945.5372,783.4846,621.4351,459.3810,221.0649 | Saponins | PPD |
| M252 | 22.37 | 1150.6135 | 1150.61429 | C56 H94 O24 | 0.69 | [M+NH_4_]^+^ | PPD-(Glc-Glc)-Glc-Glc-Ac | 425.3785,407.3685 | Saponins | PPD |
| M253 | 22.43 | 1296.63503 | 1296.62879 | C61 H100 O29 | -4.81 | [M-H]^-^ | PPD-Glc-Glc-(Glc-Xyl)-Xyl-Mal | 1209.6175,1191.6076,1077.5771,1059.5679,945.5497,783.4914,191.0548 | Saponins | PPD |
| M254 | 22.47 | 1194.60333 | 1194.6075 | C57 H94 O26 | 3.49 | [M+NH_4_]^+^ | PPD-Glc-Glc-Glc-Glc-Mal | 425.3790,407.3667 | Saponins | PPD |
| M255 | 22.49 | 1232.61898 | 1232.61493 | C60 H96 O26 | -3.29 | [M-H]^-^ | PPD-Glc-Glc-(Glc-Xyl)-Butenoyl-Mal | 1119.5857,1077.5784,1059.5693,945.5324,7834832,459.3804,191.0549 | Saponins | PPD |
| M256 | 22.50 | 1164.59277 | 1164.58875 | C56 H92 O25 | -3.45 | [M-H]^-^ | PPD-20-(Glc-Xyl)-3-(Glc-Glc)-Mal | 1077.5772,1059.5671,945.5366,783.4855,621.4339,459.3821,221.0653,191.0549 | Saponins | PPD |
| M257 | 22.50 | 1120.60294 | 1120.60117 | C55 H92 O23 | -1.58 | [M-H]^-^ | PPD-Glc-Glc-(Glc-Xyl)-Ac | 1077.5786,1059.5687,945.5387,783.4867,621.4337,191.0548 | Saponins | PPD |
| M258 | 22.60 | 956.49808 | 956.49493 | C48 H76 O19 | -3.29 | [M-H]^-^ | OA-3-GluA-Glc-28-Glc | 955.4844,793.4330,569.3811,455.3502,221.0645 | Saponins | OA |
| M259 | 22.63 | 842.50277 | 842.50217 | C44 H74 O15 | -0.71 | [M+FA-H]^-^ | OT-(Glc-Rha)-Ac | 841.4901,799.4804,653.4228,491.3708,205.0702 | Saponins | OT |
| M260 | 22.67 | 926.48752 | 926.48453 | C47 H74 O18 | -3.23 | [M-H]^-^ | OA-GluA-Glc-Xyl | 925.4746,763.4233,569.3815,455.3498 | Saponins | OA |
| M261 | 22.72 | 1262.62955 | 1262.62654 | C61 H98 O27 | -2.38 | [M-H]^-^ | PPD-(Glc-Glc)-Glc-Glc-Butenoyl-Mal | 1107.5898,945.5374,783.4888,221.0649 | Saponins | PPD |
| M262 | 22.74 | 1280.60373 | 1280.60095 | C60 H96 O29 | -2.17 | [M-H]^-^ | PPD-(Glc-Glc)-Glc-Glc-2Mal | 1149.5983,1107.5876,1089.5775,945.5370,783.4857,621.4339,459.3822,221.0654 | Saponins | PPD |
| M263 | 22.81 | 1078.5908 | 1078.58726 | C53 H90 O22 | -3.28 | [M+FA-H]^-^ | Ginsenoside Rb_2_* | 1077.5772,945.5369,783.4856,621.4340,459.3821,221.0654,191.0549 | Saponins | PPD |
| M264 | 22.83 | 1150.6135 | 1150.60781 | C56 H94 O24 | -4.95 | [M+FA-H]^-^ | PPD-(Glc-Glc)-Glc-Glc-Ac | 1107.5876,1089.5772,945.5371,783.4857,621.4337,459.3822,221.0654 | Saponins | PPD |
| M265 | 22.84 | 1234.63463 | 1234.62861 | C60 H98 O26 | -4.88 | [M-H]^-^ | PPD-(Glc-Glc)-Glc-Glc-Ac-Ac-Ac | 1233.6209,1107.5902,945.5401,783.4838,491.3338,221.0645 | Saponins | PPD |
| M266 | 22.90 | 1194.60333 | 1194.60002 | C57 H94 O26 | -2.77 | [M-H]^-^ | PPD-20-(Glc-Glc)-3-Glc-Glc-Mal | 1107.5878,1089.5777,945.5375,783.4860,621.4344,459.3824,221.0655 | Saponins | PPD |
| M267 | 22.97 | 828.48712 | 828.48731 | C43 H72 O15 | 0.23 | [M+FA-H]^-^ | OT-(Glc-Xyl)-Ac | 827.4752,785.4658,653.4244,491.3728,191.0549 | Saponins | OT |
| M268 | 23.08 | 1164.59277 | 1164.59012 | C56 H92 O25 | -2.28 | [M-H]^-^ | PPD-(Glc-Glc)-(Glc-Xyl)-Mal | 1119.5924,1077.5789,945.5387,783.4861,621.4341,459.3796,191.0547 | Saponins | PPD |
| M269 | 23.09 | 1042.49847 | 1042.49431 | C51 H78 O22 | -3.99 | [M-H]^-^ | OA-GluA-Glc-Glc-Mal | 997.4941,835.4431,613.3709,569.3807,455.3501 | Saponins | OA |
| M270 | 23.16 | 1078.5908 | 1078.58782 | C53 H90 O22 | -2.76 | [M+FA-H]^-^ | Ginsenoside Rb_3_* | 1077.5783,945.5377,783.4857,621.4340,459.3824,221.0651 | Saponins | PPD |
| M271 | 23.24 | 330.24062 | 330.23931 | C18 H34 O5 | -3.97 | [M-H]^-^ | (15Z)-9,12,13-Trihydroxy-15-octadecenoic acid | 329.23448,201.1116,171.1012,139.1116,127.1116 | Lipids | Fatty acids and derivatives |
| M272 | 23.27 | 1150.6135 | 1150.60837 | C56 H94 O24 | -4.46 | [M+FA-H]^-^ | PPD-(Glc-Glc)-Glc-Glc-Ac | 1107.5871,1089.5771,945.5367,783.4855,621.4339,459.3824,221.0653 | Saponins | PPD |
| M273 | 23.27 | 1194.60333 | 1194.59794 | C57 H94 O26 | -4.51 | [M-H]^-^ | PPD-(Glc-Glc)-Glc-Glc-Mal | 1107.5881,1089.5780,945.5375,783.4861,621.4343,459.3825,221.0655 | Saponins | PPD |
| M274 | 23.33 | 926.48752 | 926.48432 | C47 H74 O18 | -3.45 | [M-H]^-^ | OA-GluA-Glc-Xyl | 925.4744,763.4228,569.3815,455.3501 | Saponins | OA |
| M275 | 23.35 | 1236.6139 | 1236.61246 | C59 H96 O27 | -1.16 | [M-H]^-^ | PPD-(Glc-Glc)-Glc-Glc-Mal-Ac | 1149.5977,1107.5871,1089.5769,945.5377,783.4850,621.4318,459.3788,221.0648 | Saponins | PPD |
| M276 | 23.36 | 684.44218 | 684.4405 | C36 H62 O9 | -2.45 | [M+FA-H]^-^ | Ginsenoside F_1_* | 475.3772 | Saponins | PPT |
| M277 | 23.46 | 1120.60294 | 1120.59819 | C55 H92 O23 | -4.24 | [M+FA-H]^-^ | PPD-Glc-Glc-(Glc-Xyl)-Ac | 1119.5880,1077.5791,1059.5701,945.5369,783.4842,621.4329,191.0547 | Saponins | PPD |
| M278 | 23.46 | 1164.59277 | 1164.58779 | C56 H92 O25 | -4.28 | [M-H]^-^ | PPD-20-(Glc-Xyl)-3-(Glc-Glc)-Mal | 1077.5792,1059.5705,945.5388,783.4847,221.0648,191.0551 | Saponins | PPD |
| M279 | 23.72 | 994.51373 | 994.50991 | C51 H78 O19 | -3.84 | [M-H]^-^ | OA-GluA-Glc-Xyl-Butenoyl | 925.4751,785.4066,569.3820 | Saponins | OA |
| M280 | 23.72 | 926.48752 | 926.48465 | C47 H74 O18 | -3.10 | [M-H]^-^ | OA-3-GluA-Xyl-28-Glc | 925.4746,793.4340,569.3818,455.3506 | Saponins | OA |
| M281 | 23.77 | 1250.59316 | 1250.59013 | C59 H94 O28 | -2.42 | [M-H]^-^ | PPD-Glc-Glc-(Glc-Xyl)-2Mal | 1119.5841,1077.5776,1059.5678,945.5368,783.4814,621.4334,459.3812,191.0548 | Saponins | PPD |
| M282 | 23.80 | 1164.59277 | 1164.59002 | C56 H92 O25 | -2.36 | [M-H]^-^ | PPD-20-(Glc-Xyl)-3-(Glc-Glc)-Mal | 1077.5778,1059.5681,945.5373,783.4859,621.4341,459.3822,375.2887,191.0549 | Saponins | PPD |
| M283 | 23.89 | 1120.60294 | 1120.59831 | C55 H92 O23 | -4.13 | [M+FA-H]^-^ | PPD-Glc-Glc-Glc-Xyl-Ac | 1119.5880,1077.5791,1059.5701,945.5369,783.4842,621.4329 | Saponins | PPD |
| M284 | 23.91 | 1164.59277 | 1164.58777 | C56 H92 O25 | -4.29 | [M-H]^-^ | PPD-20-(Glc-Glc)-Mal-3-(Glc-Xyl) | 1077.5773,1059.5672,945.5367,783.4856,621.4339,459.3823,221.0643,191.0544 | Saponins | PPD |
| M285 | 24.04 | 1042.49847 | 1042.4935 | C51 H78 O22 | -4.77 | [M-H]^-^ | OA-GluA-Glc-Glc-Mal | 997.4957,793.4335,659.3819 | Saponins | OA |
| M286 | 24.04 | 1150.6135 | 1150.61125 | C56 H94 O24 | -1.96 | [M+FA-H]^-^ | PPD-(Glc-Glc)-Glc-Glc-Ac | 1107.5866,1089.5767,945.5365,783.4853,621.4338,459.3821,375.2883,221.0653 | Saponins | PPD |
| M287 | 24.16 | 1280.60373 | 1280.59863 | C60 H96 O29 | -3.98 | [M-H]^-^ | PPD-(Glc-Glc)-Glc-Glc-2Mal | 1149.5969,1107.5877,1089.5772,945.5377,783.4852,621.4336,459.3822,221.0649 | Saponins | PPD |
| M288 | 24.30 | 1164.59277 | 1164.58793 | C56 H92 O25 | -4.16 | [M-H]^-^ | PPD-(Glc-Glc)-(Glc-Xyl)-Mal | 1077.5777,1059.5679,945.5358,783.4855,621.4332,459.3820,221.0646,191.0544 | Saponins | PPD |
| M289 | 24.38 | 930.5552 | 930.55373 | C48 H82 O17 | -1.58 | [M+FA-H]^-^ | 443^#^-(Glc-Glc)-Glc | 929.5423,767.4905,605.4390,221.0647 | Saponins | Others |
| M290 | 24.73 | 946.55012 | 946.54857 | C48 H82 O18 | -1.64 | [M+FA-H]^-^ | PPD-20-Glc-3-Glc-Glc | 945.5364,783.4860,621.4337 | Saponins | PPD |
| M291 | 24.80 | 862.43106 | 862.42852 | C46 H70 O15 | -2.95 | [M-H]^-^ | OA-GluA-Glc-Butenoyl | 793.4336,631.3814,569.3823,455.3497 | Saponins | OA |
| M292 | 24.81 | 794.44526 | 794.44181 | C42 H66 O14 | -4.34 | [M-H]^-^ | Chikusetsusaponin Iva* | 793.4333,631.3817,569.3817,455.3506 | Saponins | OA |
| M293 | 24.81 | 862.43386 | 862.43401 | C46 H70 O15 | 0.17 | [M+FA-H]^-^ | OA-GluA-Glc-Butenoyl | 793.4359 | Saponins | OA |
| M294 | 24.83 | 1108.60294 | 1108.59994 | C54 H92 O23 | -2.71 | [M+FA-H]^-^ | PPD-(Glc-Glc)-Glc-Glc | 1107.5891,945.5383,221.0653 | Saponins | PPD |
| M295 | 24.84 | 1150.6135 | 1150.61201 | C56 H94 O24 | -1.29 | [M+FA-H]^-^ | PPD-(Glc-Glc)-Glc-Glc-Ac | 1107.5887,1089.5793,945.5349,783.4860,221.0652 | Saponins | PPD |
| M296 | 24.90 | 1164.59277 | 1164.58924 | C56 H92 O25 | -3.03 | [M-H]^-^ | PPD-Glc-Glc-(Glc-Xyl)-Mal | 1077.5788,1059.5697,945.5406,783.4880,621.4398,191.0548 | Saponins | PPD |
| M297 | 24.94 | 992.55559 | 992.55119 | C49 H84 O20 | -4.43 | [M+FA-H]^-^ | Ginsenoside Rd* | 945.5364,783.4860,621.4337,459.3827,375.2890 | Saponins | PPD |
| M298 | 24.95 | 1014.57633 | 1014.57828 | C52 H86 O19 | 1.92 | [M+FA-H]^-^ | PPD-Glc-Glc-Glc-Butenoyl | 945.5379,783.4866,621.4354 | Saponins | PPD |
| M299 | 25.18 | 1120.60294 | 1120.60064 | C55 H92 O23 | -2.05 | [M+FA-H]^-^ | PPD-Glc-Glc-(Glc-Xyl)-Ac | 1077.5777,1059.5679,945.5390,783.4859,621.4333,459.3815,191.0544 | Saponins | PPD |
| M300 | 25.26 | 1164.59277 | 1164.58928 | C56 H92 O25 | -3.00 | [M-H]^-^ | PPD-(Glc-Glc)-(Glc-Xyl)-Mal | 1077.5778,1059.5676,945.5372,783.4854,621.4338,459.3809,221.0651,191.0545 | Saponins | PPD |
| M301 | 25.33 | 1236.6139 | 1236.60896 | C59 H96 O27 | -3.99 | [M-H]^-^ | PPD-(Glc-Glc)-Glc-Glc-Mal-Ac | 1149.5977,1107.5881,1089.5783,945.5367,783.4886,621.4343,221.0653 | Saponins | PPD |
| M302 | 25.46 | 1016.55559 | 1016.55052 | C51 H84 O20 | -4.99 | [M-H]^-^ | PPD-Glc-Glc-Rha-Mal | 929.5432,767.4895,749.4817,605.4335 | Saponins | PPD |
| M303 | 25.62 | 1032.55051 | 1032.54562 | C51 H84 O21 | -4.74 | [M-H]^-^ | PPD-20-Glc-3-(Glc-Glc)-Mal | 945.53577,783.4851,621.4336,459.3820,221.06537 | Saponins | PPD |
| M304 | 25.62 | 988.56068 | 988.5582 | C50 H84 O19 | -2.51 | [M-H]^-^ | PPD-Glc-Glc-Glc-Ac | 945.5369,783.48529,621.4338,459.3814 | Saponins | PPD |
| M305 | 25.64 | 1100.57672 | 1100.57263 | C55 H88 O22 | -3.72 | [M-H]^-^ | PPD-Glc-Glc-Glc-Mal-Butenoyl | 945.5372,783.48553,621.4339 | Saponins | PPD |
| M306 | 26.00 | 988.56068 | 988.55768 | C50 H84 O19 | -3.03 | [M-H]^-^ | PPD-(Glc-Glc)-Glc-Ac | 945.5379,783.4888,621.4368,221.06536 | Saponins | PPD |
| M307 | 26.01 | 1032.55051 | 1032.54893 | C51 H84 O21 | -1.53 | [M-H]^-^ | PPD-20-Glc-Mal-3-(Glc-Glc) | 987.5479,945.5374,927.5270,7834857,621.4339,459.3817 | Saponins | PPD |
| M308 | 26.24 | 1150.6135 | 1150.61031 | C56 H94 O24 | -2.77 | [M+FA-H]^-^ | PPD-(Glc-Glc)-Glc-Glc-Ac | 1107.5874,1089.5772,945.5366,783.4839,621.4351,221.0649 | Saponins | PPD |
| M309 | 26.68 | 1118.5509 | 1118.54674 | C54 H86 O24 | -3.72 | [M-H]^-^ | PPD-20-Glc-Mal-3-(Glc-Glc)-Mal | 987.5456,945.5376,927.5263,783.4840,621.4340 | Saponins | PPD |
| M310 | 26.69 | 1074.56107 | 1074.55836 | C53 H86 O22 | -2.52 | [M-H]^-^ | PPD-Glc-Glc-Glc-Mal-Ac | 987.5501,945.5385,927.5273,783.4845,621.4338 | Saponins | PPD |
| M311 | 26.76 | 1032.55051 | 1032.54794 | C51 H84 O21 | -2.49 | [M-H]^-^ | PPD-20-Glc-3-(Glc-Glc)-Mal | 945.5378,783.4818,621.4357 | Saponins | PPD |
| M312 | 26.83 | 1176.62915 | 1176.62772 | C58 H96 O24 | -1.22 | [M+FA-H]^-^ | PPD-20-(Glc-Glc)-3-Glc-Glc-Butenoyl | 1107.5884,1089.5781,945.5377,783.4860,621.4342,459.3825,221.0654 | Saponins | PPD |
| M313 | 26.89 | 946.55012 | 946.54581 | C48 H82 O18 | -4.55 | [M+FA-H]^-^ | Gypenoside XVII* | 945.5357,783.4848,621.4337,459.3820,221.0654 | Saponins | PPD |
| M314 | 27.01 | 1118.5509 | 1118.54781 | C54 H86 O24 | -2.76 | [M-H]^-^ | PPD-(Glc-Glc)-Glc-2Mal | 987.5486,945.5383,783.4895,621.4318,459.3815,221.0656 | Saponins | PPD |
| M315 | 27.06 | 988.56068 | 988.55753 | C50 H84 O19 | -3.19 | [M+FA-H]^-^ | PPD-(Glc-Glc)-Glc-Ac | 987.5470,945.5372,927.5272,783.4851,621.4337,459.3819 | Saponins | PPD |
| M316 | 27.15 | 988.56068 | 988.5565 | C50 H84 O19 | -4.23 | [M-H]^-^ | PPD-Glc-Glc-Glc-Ac | 945.5384,783.4866,621.4335,459.3825 | Saponins | PPD |
| M317 | 27.15 | 1032.55051 | 1032.54631 | C51 H84 O21 | -4.07 | [M-H]^-^ | PPD-20-Glc-3-(Glc-Glc)-Mal | 945.5368,783.4854,621.4336,459.3821,375.2881,221.0656 | Saponins | PPD |
| M318 | 27.46 | 970.47734 | 970.47254 | C48 H74 O20 | -4.95 | [M+NH_4_]^+^ | OA-GluA-GluA-Glc | 439.3589 | Saponins | OA |
| M319 | 27.53 | 1032.55051 | 1032.54713 | C51 H84 O21 | -3.27 | [M-H]^-^ | PPD-(Glc-Glc)-Glc-Mal | 945.5369,7834854,621.4340,459.3821,375.2887,221.0650 | Saponins | PPD |
| M320 | 27.63 | 1118.5509 | 1118.54623 | C54 H86 O24 | -4.18 | [M-H]^-^ | PPD-(Glc-Glc)-Glc-2Mal | 987.5523,945.5381,927.5276,783.4857,621.4348,459.3814,221.0652 | Saponins | PPD |
| M321 | 27.79 | 1074.56107 | 1074.55787 | C53 H86 O22 | -2.98 | [M-H]^-^ | PPD-20-Glc-3-(Glc-Glc)-Mal-Ac | 987.5496,945.5386,783.4787,621.4332,459.3818,221.0655 | Saponins | PPD |
| M322 | 27.90 | 1146.61859 | 1146.61579 | C57 H94 O23 | -2.44 | [M+FA-H]^-^ | PPD-Glc-Glc-(Glc-Xyl)-Butenoyl | 1077.5791,1059.5683,945.5394,783.4880,621.4335,191.0547 | Saponins | PPD |
| M323 | 27.91 | 1118.5509 | 1118.54629 | C54 H86 O24 | -4.12 | [M-H]^-^ | PPD-20-Glc-Mal-3-(Glc-Glc)-Mal | 987.5510,945.5383,927.5282,783.4882,621.4346 | Saponins | PPD |
| M324 | 28.05 | 1032.55051 | 1032.54657 | C51 H84 O21 | -3.82 | [M-H]^-^ | PPD-20-(Glc-Glc)-Mal-3-Glc | 945.5378,927.5277,783.4888,621.4343,459.3826,221.0651 | Saponins | PPD |
| M325 | 28.08 | 916.53955 | 916.53859 | C47 H80 O17 | -1.05 | [M+FA-H]^-^ | PPD-20-(Glc-Xyl)-3-Glc | 915.5300,783.4884,621.4354,459.3840,191.0548 | Saponins | PPD |
| M326 | 28.21 | 916.53955 | 916.53835 | C47 H80 O17 | -1.31 | [M+FA-H]^-^ | PPD-Glc-(Glc-Xyl) | 915.5271,783.4856,621.4339,459.3820,191.0545 | Saponins | PPD |
| M327 | 28.28 | 1032.55051 | 1032.54762 | C51 H84 O21 | -2.80 | [M-H]^-^ | PPD-20-(Glc-Glc)-Mal-3-Glc | 945.5373,783.4861,621.4337,459.3829,221.0649 | Saponins | PPD |
| M328 | 28.32 | 988.56068 | 988.55728 | C50 H84 O19 | -3.44 | [M+Cl]^-^ | PPD-20-Glc-3-Glc-Glc-Ac | 945.5358 | Saponins | PPD |
| M329 | 28.35 | 916.53955 | 916.53883 | C47 H80 O17 | -0.79 | [M+FA-H]^-^ | PPD-(Glc-Glc)-Xyl | 915.5264,783.4850,621.4335,459.3814,375.2889,221.0653 | Saponins | PPD |
| M330 | 28.39 | 1176.62915 | 1176.62665 | C58 H96 O24 | -2.12 | [M+FA-H]^-^ | PPD-(Glc-Glc)-Glc-Glc-Butenoyl | 1107.5881,945.5371,783.4862,621.4375,459.3818,221.0648 | Saponins | PPD |
| M331 | 28.61 | 1002.53994 | 1002.53511 | C50 H82 O20 | -4.82 | [M-H]^-^ | PPD-20-Xyl-3-(Glc-Glc)-Mal | 915.5265,783.4852,621.4336,459.3818,375.2879,221.0650 | Saponins | PPD |
| M332 | 28.73 | 1002.53994 | 1002.53921 | C50 H82 O20 | -0.73 | [M-H]^-^ | PPD-20-Xyl-3-(Glc-Glc)-Mal | 915.5266,783.4856,621.4337,459.3816,375.2876,221.0651 | Saponins | PPD |
| M333 | 28.77 | 988.56068 | 988.55728 | C50 H84 O19 | -3.44 | [M+FA-H]^-^ | PPD-20-(Glc-Glc)-3-Glc-Ac | 945.5382,927.5239,783.4846,621.4367,459.3843,,221.0650 | Saponins | PPD |
| M334 | 28.81 | 988.56068 | 988.56466 | C50 H84 O19 | 4.03 | [M+NH_4_]^+^ | PPD-Glc-Glc-Glc-Ac | 407.3686 | Saponins | PPD |
| M335 | 28.91 | 916.53955 | 916.53883 | C47 H80 O17 | -0.79 | [M+FA-H]^-^ | PPD-20-(Glc-Xyl)-3-Glc | 915.5294,783.4819,621.4351 | Saponins | PPD |
| M336 | 29.24 | 988.56068 | 988.55934 | C50 H84 O19 | -1.36 | [M+FA-H]^-^ | PPD-Glc-Glc-Glc-Ac | 987.5480,945.5391,927.5297,783.4884,621.4330 | Saponins | PPD |
| M337 | 29.37 | 766.48673 | 766.48733 | C42 H70 O12 | 0.78 | [M+FA-H]^-^ | Ginsenoside F_4_* | 765.4766,619.4184 | Saponins | PPT |
| M338 | 29.39 | 1002.53994 | 1002.53658 | C50 H82 O20 | -3.35 | [M-H]^-^ | PPD-Glc-Glc-Xyl-Mal | 915.5276,783.4859,621.4343,459.3822 | Saponins | PPD |
| M339 | 29.47 | 492.38147 | 492.38218 | C30 H52 O5 | 1.44 | [M+H]^+^ | Ocotillol | 439.3599 | Saponins | OT |
| M340 | 29.76 | 930.5552 | 930.55439 | C48 H82 O17 | -0.87 | [M+FA-H]^-^ | PPD-20-Rha-3-Glc-Glc | 929.5435,783.4868,621.4333,459.3808 | Saponins | PPD |
| M341 | 29.91 | 1014.57633 | 1014.5751 | C52 H86 O19 | -1.21 | [M+FA-H]^-^ | PPD-20-Glc-3-Glc-Glc-Butenoyl | 945.5371,783.4857,621.4340,459.3826 | Saponins | PPD |
| M342 | 30.01 | 1016.55559 | 1016.55096 | C51 H84 O20 | -4.55 | [M-H]^-^ | PPD-Glc-Glc-Rha-Mal | 929.5436,783.4891,621.4343,459.3822 | Saponins | PPD |
| M343 | 30.12 | 1016.55559 | 1016.55123 | C51 H84 O20 | -4.29 | [M-H]^-^ | PPD-(Glc-Glc)-Rha-Mal | 929.5423,783.4853,621.4341,459.3817,375.2877 | Saponins | PPD |
| M344 | 30.27 | 830.50277 | 830.49882 | C43 H74 O15 | -4.76 | [M+FA-H]^-^ | Ginsenoside F_2_* | 783.4877,621.4337,459.3822 | Saponins | PPD |
| M345 | 30.46 | 794.44526 | 794.44282 | C42 H66 O14 | -3.07 | [M-H]^-^ | OA-3-GluA-Glc | 793.4337,569.3826,455.3512 | Saponins | OA |
| M346 | 30.65 | 926.48752 | 926.48545 | C47 H74 O18 | -2.23 | [M+NH_4_]^+^ | OA-GluA-Glc-Xyl | 439.3586,393.3530 | Saponins | OA |
| M347 | 30.66 | 826.50786 | 826.50405 | C44 H74 O14 | -4.61 | [M-H]^-^ | PPD-Glc-Glc-Ac | 621.4339,459.3817 | Saponins | PPD |
| M348 | 30.66 | 870.49769 | 870.49371 | C45 H74 O16 | -4.57 | [M-H]^-^ | PPD-20-Glc-Mal-3-Glc | 783.4812,621.4340,459.3830 | Saponins | PPD |
| M349 | 31.11 | 1232.69175 | 1232.69004 | C62 H104 O24 | -1.39 | [M+FA-H]^-^ | PPD-20-Glc-Glc-3-Glc-Glc-Octenoyl | 1107.5880,945.5375,783.4857,621.4336,459.3818,221.0649 | Saponins | PPD |
| M350 | 31.17 | 870.49769 | 870.49404 | C45 H74 O16 | -4.19 | [M-H]^-^ | PPD-20-Glc-3-Glc-Mal | 783.4865 | Saponins | PPD |
| M351 | 31.39 | 784.49729 | 784.49575 | C42 H72 O13 | -1.96 | [M+FA-H]^-^ | 20(S)-Ginsenoside Rg_3_* | 783.4856,621.5337,459.3820 | Saponins | PPD |
| M352 | 31.60 | 870.49769 | 870.49337 | C45 H74 O16 | -4.96 | [M-H]^-^ | PPD-3-Glc-Glc-Mal | 825.4935,783.4860,621.4339,459.3824,375.2890 | Saponins | PPD |
| M353 | 31.65 | 784.49729 | 784.49855 | C42 H72 O13 | 1.61 | [M+FA-H]^-^ | 20(R)-Ginsenoside Rg_3_* | 783.4862,621.4365,459.3812,221.0656 | Saponins | PPD |
| M354 | 31.69 | 317.29299 | 317.29383 | C18 H39 N O3 | 2.65 | [M+H]^+^ | 2-Amino-1,3,4-octadecanetriol | 300.2907,282.2797,252.2685 | Others | Others |
| M355 | 31.88 | 490.36582 | 490.36683 | C30 H50 O5 | 2.06 | [M+H]^+^ | Pseudo-ginsengenin R_1_ (489) | 437.3413,419.3327 | Saponins | Others |
| M356 | 31.88 | 312.23006 | 312.22947 | C18 H32 O4 | -1.89 | [M-H]^-^ | (+/-)9-HpODE | 293.2114,185.1173,171.1017,139.1118 | Lipids | Fatty acids and derivatives |
| M357 | 32.17 | 764.43469 | 764.43172 | C41 H64 O13 | -3.89 | [M-H]^-^ | OA-3-GluA-Xyl | 763.4230,631.3810,569.3819 | Saponins | OA |
| M358 | 32.54 | 312.23006 | 312.22946 | C18 H32 O4 | -1.92 | [M-H]^-^ | (+/-)9-HpODE | 293.2107,201.1122,171.1013,127.1117 | Lipids | Fatty acids and derivatives |
| M359 | 32.99 | 632.39243 | 632.38937 | C36 H56 O9 | -4.84 | [M-H]^-^ | OA-GluA | 631.3811,455.3500 | Saponins | OA |
| M360 | 34.25 | 294.18311 | 294.18233 | C17 H26 O4 | -2.65 | [M-H_2_O-H]^-^ | 3-[1-Hydroxy-3,5-bis(2-methyl-2-propanyl)-4-oxo-2,5-cyclohexadien-1-yl]propanoic acid | 275.1669,231.1739 | Organic acids and derivatives | Organic acids and derivatives |
| M361 | 34.30 | 236.17763 | 236.17741 | C15 H24 O2 | -0.93 | [M-H_2_O+H]^+^ | 4-[(3E)-5-Hydroxy-3-methyl-3-penten-1-yl]-3,5,5-trimethyl-2-cyclohexen-1-one | 219.1748,189.1278,149.0964,123.1171,93.0701,81.0701 | Others | Others |
| M362 | 34.79 | 453.28554 | 453.28645 | C21 H44 N O7 P | 2.01 | [M+H]^+^ | Glycerophospho-N-palmitoyl ethanolamine | 313.2746,282.2797,109.1013 | Lipids | Glycerophospholipids |
| M363 | 35.32 | 314.24571 | 314.24461 | C18 H34 O4 | -3.50 | [M-H_2_O-H]^-^ | (+/-)12(13)-DiHOME | 295.2270,277.2157,195.1377 | Lipids | Fatty acids and derivatives |
| M364 | 35.37 | 516.32983 | 516.32731 | C27 H48 O9 | -4.88 | [M+NH_4_]^+^ | 3-(Hexopyranosyloxy)-2-hydroxypropyl (9Z,12Z)-9,12-octadecadienoate | 337.2744,263.2373,245.2271,95.0856 | Others | Others |
| M365 | 35.61 | 354.27701 | 354.27753 | C21 H38 O4 | 1.47 | [M-H_2_O+H]^+^ | 1-Linoleoyl glycerol | 337.2746,109.1014,95.0857,81.0700 | Lipids | Glycerolipids |
| M366 | 35.75 | 280.24023 | 280.24123 | C18 H32 O2 | 3.57 | [M-H_2_O+H]^+^ | octadec-9-ynoic acid | 109.1014,95.0857,81.0700,67.0544 | Lipids | Fatty acids and derivatives |
| M367 | 35.75 | 516.32983 | 516.33092 | C27 H48 O9 | 2.11 | [M+NH_4_]^+^ | 3-(Hexopyranosyloxy)-2-hydroxypropyl (9Z,12Z)-9,12-octadecadienoate | 337.2751,263.2375,245.2269,109.1013,95.0856 | Others | Others |
| M368 | 35.76 | 294.21949 | 294.21821 | C18 H30 O3 | -4.35 | [M-H]^-^ | 13(S)-HOTrE | 293.2106,192.1142,113.0962 | Lipids | Fatty acids and derivatives |
| M369 | 35.86 | 294.21949 | 294.22009 | C18 H30 O3 | 2.04 | [M+H]^+^ | 13(S)-HOTrE | 295.2096,179.1434,135.1171,107.0858,93.0699,79.0544 | Lipids | Fatty acids and derivatives |
| M370 | 36.00 | 354.27701 | 354.27753 | C21 H38 O4 | 1.47 | [M-H_2_O+H]^+^ | 1-Linoleoyl glycerol | 337.2747,263.2372,109.1013,95.0856,81.0699,67.0543 | Lipids | Glycerolipids |
| M371 | 36.00 | 280.24023 | 280.24132 | C18 H32 O2 | 3.89 | [M-H_2_O+H]^+^ | octadec-9-ynoic acid | 109.1012,95.0856,81.0699,67.0543 | Lipids | Fatty acids and derivatives |
| M372 | 36.01 | 294.21949 | 294.22036 | C18 H30 O3 | 2.96 | [M+H]^+^ | 13(S)-HOTrE | 295.2091,151.1118,107.0857,93.0699,81.0336 | Lipids | Fatty acids and derivatives |
| M373 | 36.11 | 196.10005 | 196.10066 | C13 H12 N2 | 3.11 | [M+H]^+^ | 2-ethyl-1H-perimidine | 197.1079,182.0844,154.0656 | Others | Others |
| M374 | 36.12 | 294.21949 | 294.22046 | C18 H30 O3 | 3.30 | [M+H]^+^ | 13(S)-HOTrE | 295.2091,151.1118,107.0857,93.0699,81.0336 | Lipids | Fatty acids and derivatives |
| M375 | 36.12 | 254.22458 | 254.22576 | C16 H30 O2 | 4.64 | [M+Na]^+^ | Palmitoleic Acid | 235.1697,133.1014,105.0700,95.0492,81.0335 | Lipids | Fatty acids and derivatives |
| M376 | 36.34 | 294.21949 | 294.22031 | C18 H30 O3 | 2.79 | [M+H]^+^ | 13(S)-HOTrE | 295.2099,277.2171,151.1120,95.0493,81.0336 | Lipids | Fatty acids and derivatives |
| M377 | 37.44 | 472.35526 | 472.35636 | C30 H48 O4 | 2.33 | [M+Na]^+^ | Maslinic acid | 496.3490,495.3454 | Lipids | Prenol Lipids |
| M378 | 37.93 | 436.25899 | 436.25696 | C21 H41 O7 P | -4.65 | [M-H]^-^ | Oleoyl-L-α-lysophosphatidic acid | 435.2517,281.2478,152.9948,96.9687 | Phenols | Phenols |
| M379 | 38.11 | 299.28243 | 299.28318 | C18 H37 N O2 | 2.51 | [M+H]^+^ | Sphingosine (d18:1) | 282.2796,95.0856,71.0856 | Others | Amino alcohol |
| M380 | 38.54 | 272.23514 | 272.23476 | C16 H32 O3 | -1.40 | [M-H]^-^ | 16-Hydroxyhexadecanoic acid | 271.2263,253.2158,225.2209 | Lipids | Fatty acids and derivatives |
| M381 | 40.02 | 356.23514 | 356.23625 | C23 H32 O3 | 3.12 | [M+H]^+^ | Ilicicolin B | 167.0706,109.1013,95.0856,81.0700 | Others | Others |
| M382 | 40.29 | 738.41904 | 738.41584 | C39 H62 O13 | -4.33 | [M+FA-H]^-^ | 413^#^-(Glc-Glc) | 737.4815,615.5355,221.0654 | Saponins | Others |

Note: xyl was used to represent all possible pentose moieties, including ara(*p*) and ara(*f*), which could not be discriminated by mass spectrometry.

*: Ginsenosides identified by comparison with reliable reference standards.

^#^: deprotonated ion of the sapogenins in negative model.
