# Supplementary material for: Discrimination for geographical origin of Panax quinquefolius L. using UPLC Q‐Orbitrap MS‐based metabolomics approach
Source: Food Sci Nutr. 2023 Jul 10;11(8):4843–52. doi: 10.1002/fsn3.3461 (PMC10420767; doi:10.1002/fsn3.3461)
Supplement: Supplementary file 3 — Table S3 [file FSN3-11-4843-s003.docx]

Table S3 65 shared differential metabolites were identified between UC and the other four producing areas

| No. | RT [min] | Theoretical Mass (Da) | Measured Mass (Da) | Formula | Error  [ppm] | Ionization  model | Identification compound | Class Ⅰ | Class Ⅱ |
| --- | --- | --- | --- | --- | --- | --- | --- | --- | --- |
| M1 | 0.82 | 146.10553 | 146.10592 | C6 H14 N2 O2 | 2.67 | [M+H]^+^ | Lysine* | Amino acids and derivatives | Amino acids and derivatives |
| M8 | 0.93 | 146.06914 | 146.06972 | C5 H10 N2 O3 | 3.97 | [M+H]^+^ | DL-Glutamine | Amino acids and derivatives | Amino acids and derivatives |
| M11 | 0.97 | 147.05316 | 147.05377 | C5 H9 N O4 | 4.15 | [M+H]^+^ | Glutamic acid* | Amino acids and derivatives | Amino acids and derivatives |
| M16 | 1.01 | 290.12263 | 290.12372 | C10 H18 N4 O6 | 3.76 | [M+H]^+^ | Argininosuccinic acid | Amino acids and derivatives | Amino acids and derivatives |
| M38 | 1.69 | 149.05105 | 149.05148 | C5 H11 N O2 S | 2.88 | [M+H]^+^ | L-(-)-Methionine* | Amino acids and derivatives | Amino acids and derivatives |
| M45 | 2.30 | 116.01096 | 116.01046 | C4 H4 O4 | -4.31 | [M-H]^-^ | Fumaric acid* | Organic acids and derivatives | Organic acids and derivatives |
| M49 | 2.83 | 244.06954 | 244.06896 | C9 H12 N2 O6 | -2.38 | [M-H]^-^ | Uridine | Nucleotides and derivatives | Ribonucleoside |
| M50 | 2.84 | 131.09463 | 131.09497 | C6 H13 N O2 | 2.59 | [M+H]^+^ | leucine* | Amino acids and derivatives | Amino acids and derivatives |
| M56 | 5.11 | 267.09675 | 267.09722 | C10 H13 N5 O4 | 1.76 | [M+H]^+^ | Adenosine | Nucleotides and derivatives | Ribonucleoside |
| M57 | 6.53 | 283.09167 | 283.09111 | C10 H13 N5 O5 | -1.98 | [M-H]^-^ | Guanosine | Nucleotides and derivatives | Ribonucleoside |
| M58 | 6.55 | 151.04941 | 151.04977 | C5 H5 N5 O | 2.38 | [M+H]^+^ | Guanine | Nucleotides and derivatives | purines |
| M59 | 6.85 | 165.07898 | 165.07937 | C9 H11 N O2 | 2.36 | [M+H]^+^ | L-Phenylalanine* | Amino acids and derivatives | Amino acids and derivatives |
| M61 | 7.08 | 284.07568 | 284.07466 | C10 H12 N4 O6 | -3.59 | [M-H]^-^ | Xanthosine | Nucleotides and derivatives | Ribonucleoside |
| M63 | 7.25 | 297.10732 | 297.10687 | C11 H15 N5 O5 | -1.51 | [M-H]^-^ | 2'-O-Methylguanosine | Nucleotides and derivatives | Ribonucleoside |
| M70 | 7.64 | 244.16746 | 244.16704 | C13 H24 O4 | -1.72 | [M+Cl]^-^ | 4-(Hydroxymethyl)-3-(1-hydroxyoctyl)dihydro-2(3H)-furanone | Carbohydrates and derivatives | Carbohydrates and derivatives |
| M72 | 8.05 | 297.08956 | 297.09026 | C11 H15 N5 O3 S | 2.36 | [M+H]^+^ | 5'-S-Methyl-5'-thioadenosine | Others | Others |
| M78 | 8.62 | 176.06847 | 176.06809 | C7 H12 O5 | -2.16 | [M-H]^-^ | 2-Isopropylmalic acid | Organic acids and derivatives | Organic acids and derivatives |
| M80 | 8.67 | 180.04226 | 180.04205 | C9 H8 O4 | -1.17 | [M-H_2_O+H]^+^ | 3-Hydroxy-7-methoxy-2-benzofuran-1(3H)-one | Others | Others |
| M94 | 9.23 | 180.04226 | 180.04178 | C9 H8 O4 | -2.67 | [M-H]^-^ | Caffeic acid | Organic acids and derivatives | Organic acids and derivatives |
| M95 | 9.29 | 328.14231 | 328.14319 | C18 H20 N2 O4 | 2.68 | [M+H]^+^ | N1-(2-{2-[2-(acetylamino)phenoxy]ethoxy}phenyl)acetamide | Others | Carboxamides |
| M101 | 9.50 | 948.56577 | 948.56456 | C48 H84 O18 | -1.28 | [M+FA-H]^-^ | 477^#^-Glc-Glc-Rha | Saponins | Others |
| M115 | 9.87 | 1124.59785 | 1124.59307 | C54 H92 O24 | -4.25 | [M+FA-H]^-^ | OT-Glc-Glc-Glc-Rha | Saponins | OT |
| M116 | 10.00 | 960.49299 | 960.49726 | C47 H76 O20 | 4.45 | [M+FA-H]^-^ | 489^#^-Glc-Glc-Rha | Saponins | Others |
| M131 | 10.77 | 1108.60294 | 1108.59925 | C54 H92 O23 | -3.33 | [M+FA-H]^-^ | PPT-(Glc-Glc)-Glc-Rha | Saponins | PPT |
| M133 | 10.83 | 962.54503 | 962.54342 | C48 H82 O19 | -1.67 | [M+FA-H]^-^ | PPT-(Glc-Glc)-Glc | Saponins | PPT |
| M136 | 10.92 | 1108.60294 | 1108.59924 | C54 H92 O23 | -3.34 | [M+FA-H]^-^ | PPT-(Glc-Glc)-Glc-Rha | Saponins | PPT |
| M142 | 11.36 | 1108.60294 | 1108.60083 | C54 H92 O23 | -1.90 | [M+FA-H]^-^ | PPT-(Glc-Glc)-Glc-Rha | Saponins | PPT |
| M147 | 11.61 | 962.54503 | 962.5432 | C48 H82 O19 | -1.90 | [M+FA-H]^-^ | PPT-Glc-Glc-Glc | Saponins | PPT |
| M148 | 11.61 | 188.10486 | 188.10397 | C9 H16 O4 | -4.73 | [M-H]^-^ | Azelaic acid | Lipids | Fatty acids and derivatives |
| M150 | 11.82 | 1108.60294 | 1108.60047 | C54 H92 O23 | -2.23 | [M+FA-H]^-^ | PPT-Glc-Glc-Glc-Rha | Saponins | PPT |
| M152 | 11.85 | 962.54503 | 962.54322 | C48 H82 O19 | -1.88 | [M+FA-H]^-^ | PPT-Glc-Glc-Glc | Saponins | PPT |
| M175 | 13.92 | 962.54503 | 962.54288 | C48 H82 O19 | -2.23 | [M+FA-H]^-^ | OT-Glc-Glc-Rha | Saponins | OT |
| M178 | 14.20 | 1124.59785 | 1124.59422 | C54 H92 O24 | -3.23 | [M+FA-H]^-^ | PPT-(Glc-Glc)-Glc-Glc | Saponins | PPT |
| M209 | 18.18 | 1270.65576 | 1270.65104 | C60 H102 O28 | -3.71 | [M+FA-H]^-^ | PPD-(Glc-Glc)-Glc-Glc-Glc | Saponins | PPD |
| M210 | 18.52 | 1356.65616 | 1356.64947 | C63 H104 O31 | -4.93 | [M-H]^-^ | PPD-(Glc-Glc)-Glc-Glc-Glc-Mal | Saponins | PPD |
| M212 | 18.84 | 1356.65616 | 1356.64947 | C63 H104 O31 | -4.93 | [M-H]^-^ | PPD-(Glc-Glc)-Glc-Glc-Glc-Mal | Saponins | PPD |
| M215 | 19.30 | 1356.65616 | 1356.64995 | C63 H104 O31 | -4.58 | [M-H]^-^ | PPD-(Glc-Glc)-Glc-Glc-Glc-Mal | Saponins | PPD |
| M217 | 19.35 | 1270.65576 | 1270.64975 | C60 H102 O28 | -4.73 | [M+FA-H]^-^ | PPD-(Glc-Glc)-Glc-Glc-Glc | Saponins | PPD |
| M223 | 20.02 | 1356.65616 | 1356.64986 | C63 H104 O31 | -4.64 | [M-H]^-^ | PPD-(Glc-Glc)-Glc-Glc-Glc-Mal | Saponins | PPD |
| M226 | 20.17 | 1270.65576 | 1270.65147 | C60 H102 O28 | -3.38 | [M+FA-H]^-^ | PPD-(Glc-Glc)-Glc-Glc-Glc | Saponins | PPD |
| M232 | 20.59 | 1194.60333 | 1194.59839 | C57 H94 O26 | -4.14 | [M-H]^-^ | PPD-(Glc-Glc)-Glc-Glc-Mal | Saponins | PPD |
| M252 | 22.37 | 1150.6135 | 1150.61429 | C56 H94 O24 | 0.69 | [M+NH_4_]^+^ | PPD-(Glc-Glc)-Glc-Glc-Ac | Saponins | PPD |
| M286 | 24.04 | 1150.6135 | 1150.61125 | C56 H94 O24 | -1.96 | [M+FA-H]^-^ | PPD-(Glc-Glc)-Glc-Glc-Ac | Saponins | PPD |
| M294 | 24.83 | 1108.60294 | 1108.59994 | C54 H92 O23 | -2.71 | [M+FA-H]^-^ | PPD-(Glc-Glc)-Glc-Glc | Saponins | PPD |
| M295 | 24.84 | 1150.6135 | 1150.61201 | C56 H94 O24 | -1.29 | [M+FA-H]^-^ | PPD-(Glc-Glc)-Glc-Glc-Ac | Saponins | PPD |
| M301 | 25.33 | 1236.6139 | 1236.60896 | C59 H96 O27 | -3.99 | [M-H]^-^ | PPD-(Glc-Glc)-Glc-Glc-Mal-Ac | Saponins | PPD |
| M308 | 26.24 | 1150.6135 | 1150.61031 | C56 H94 O24 | -2.77 | [M+FA-H]^-^ | PPD-(Glc-Glc)-Glc-Glc-Ac | Saponins | PPD |
| M318 | 27.46 | 970.47734 | 970.47254 | C48 H74 O20 | -4.95 | [M+NH_4_]^+^ | OA-GluA-GluA-Glc | Saponins | OA |
| M328 | 28.32 | 988.56068 | 988.55728 | C50 H84 O19 | -3.44 | [M+Cl]^-^ | PPD-20-Glc-3-Glc-Glc-Ac | Saponins | PPD |
| M334 | 28.81 | 988.56068 | 988.56466 | C50 H84 O19 | 4.03 | [M+NH_4_]^+^ | PPD-Glc-Glc-Glc-Ac | Saponins | PPD |
| M336 | 29.24 | 988.56068 | 988.55934 | C50 H84 O19 | -1.36 | [M+FA-H]^-^ | PPD-Glc-Glc-Glc-Ac | Saponins | PPD |
| M345 | 30.46 | 794.44526 | 794.44282 | C42 H66 O14 | -3.07 | [M-H]^-^ | OA-3-GluA-Glc | Saponins | OA |
| M349 | 31.11 | 1232.69175 | 1232.69004 | C62 H104 O24 | -1.39 | [M+FA-H]^-^ | PPD-20-Glc-Glc-3-Glc-Glc-Octenoyl | Saponins | PPD |
| M354 | 31.69 | 317.29299 | 317.29383 | C18 H39 N O3 | 2.65 | [M+H]^+^ | 2-Amino-1,3,4-octadecanetriol | Others | Others |
| M356 | 31.88 | 312.23006 | 312.22947 | C18 H32 O4 | -1.89 | [M-H]^-^ | (+/-)9-HpODE | Lipids | Fatty acids and derivatives |
| M362 | 34.79 | 453.28554 | 453.28645 | C21 H44 N O7 P | 2.01 | [M+H]^+^ | Glycerophospho-N-palmitoyl ethanolamine | Lipids | Glycerophospholipids |
| M364 | 35.37 | 516.32983 | 516.32731 | C27 H48 O9 | -4.88 | [M+NH_4_]^+^ | 3-(Hexopyranosyloxy)-2-hydroxypropyl (9Z,12Z)-9,12-octadecadienoate | Others | Others |
| M366 | 35.75 | 280.24023 | 280.24123 | C18 H32 O2 | 3.57 | [M-H_2_O+H]^+^ | octadec-9-ynoic acid | Lipids | Fatty acids and derivatives |
| M367 | 35.75 | 516.32983 | 516.33092 | C27 H48 O9 | 2.11 | [M+NH_4_]^+^ | 3-(Hexopyranosyloxy)-2-hydroxypropyl (9Z,12Z)-9,12-octadecadienoate | Others | Others |
| M368 | 35.76 | 294.21949 | 294.21821 | C18 H30 O3 | -4.35 | [M-H]^-^ | 13(S)-HOTrE | Lipids | Fatty acids and derivatives |
| M369 | 35.86 | 294.21949 | 294.22009 | C18 H30 O3 | 2.04 | [M+H]^+^ | 13(S)-HOTrE | Lipids | Fatty acids and derivatives |
| M372 | 36.01 | 294.21949 | 294.22036 | C18 H30 O3 | 2.96 | [M+H]^+^ | 13(S)-HOTrE | Lipids | Fatty acids and derivatives |
| M376 | 36.34 | 294.21949 | 294.22031 | C18 H30 O3 | 2.79 | [M+H]^+^ | 13(S)-HOTrE | Lipids | Fatty acids and derivatives |
| M380 | 38.54 | 272.23514 | 272.23476 | C16 H32 O3 | -1.40 | [M-H]^-^ | 16-Hydroxyhexadecanoic acid | Lipids | Fatty acids and derivatives |
| M382 | 40.29 | 738.41904 | 738.41584 | C39 H62 O13 | -4.33 | [M+FA-H]^-^ | 413^#^-(Glc-Glc) | Saponins | Others |

*: Ginsenosides identified by comparison with reliable reference standards.

^#^: deprotonated ion of the sapogenins in negative model.
