# Supplementary material for: Discrimination for geographical origin of Panax quinquefolius L. using UPLC Q‐Orbitrap MS‐based metabolomics approach
Source: Food Sci Nutr. 2023 Jul 10;11(8):4843–52. doi: 10.1002/fsn3.3461 (PMC10420767; doi:10.1002/fsn3.3461)
Supplement: Supplementary file 4 — Appendix S1 [file FSN3-11-4843-s002.docx]

Supplementary Information Sections

**1. The sampling principles and design**

Canada, America, and China are the largest country for American ginseng cultivation. American ginseng from America and Canada is mainly cultivated in the border of that. We took samples from the main cultivation areas of American ginseng in Marathon in the US, and Toronto and Montreal in Canada, respectively.

American ginseng is primarily cultivated in Heilongjiang, Jilin, Liaoning, and Shandong Provinces in China. Due to continuous cropping obstacles leading land shortage suitable for ginseng growing in Jilin Province, many farmers have transferred to Heilongjiang Province for American ginseng cultivation, resulting in widespread cultivation in the region. For this study, three locations (Zhanhe, Tieli, and Raohe) were selected from west to east in central Heilongjiang Province to represent this region. American ginseng from Jilin and Liaoning Province is mainly cultivated in the eastern mountainous area. Therefore, nine locations (Jiaohe, Antu, Hunchun, Fusong, Changbai, Ji'an, Huanren, Kuandian, and Gaizhou) were selected to represent the main cultivating regions in Jilin and Liaoning Province from north to south. Weihai is the central cultivating region for American ginseng in Shandong Province. Thus, we selected Weihai and the surrounding cities of Yantai and Rongcheng, which are located to the west and east of Weihai, respectively. Overall, we collected samples from nineteen different origins across five producing areas. The distribution of the sampling points is illustrated in Figure S1.


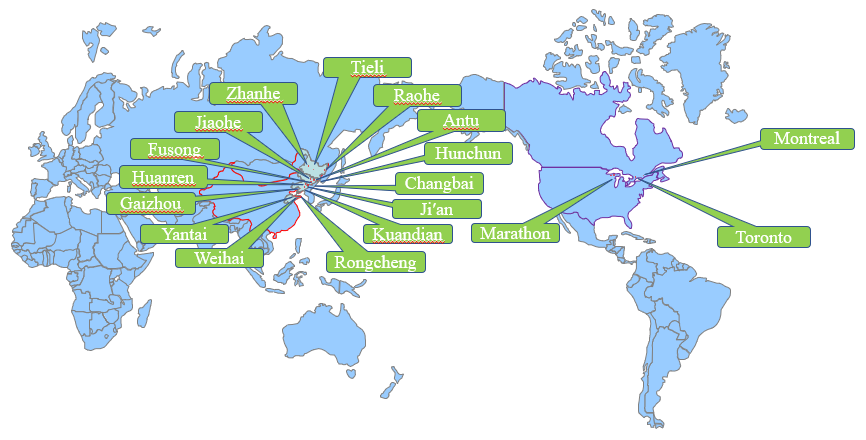


FIGURE S1 Sampling points of American ginseng

**2. Spectrum elucidation of representative saponins**

The fragmentations of ginsenosides were studied, based on previous reports (Shi et al., 2017; Wang et al., 2019; Yang et al., 2019, 2012; Zhang et al., 2020; Zuo et al., 2020). Their ESI-MS^2^ spectra provided diagnostic information for the sapogenins, sugar chains and [acyl group](javascript:;)s. In a word, in negative-mode, PPD-type, PPT-type, OA-type, and OT-type ginsenosides were characterized by the sapogenins ion at m/z 459, 475, 455, and 491, respectively. The lost masses of 162 Da, 146 Da, 132 Da, and 176 Da were ascribed to the elimination of Glc, Rha, Ara (or Xyl), and GluA residue, respectively. The lost masses of 86 Da, 68 Da, and 42 Da were attributed to the elimination of malonyl, butenoyl, and acetyl, respectively. In positive-mode, [M+Na]^+^ ion of PPD-type, PPT-type ginsenosides was easier to lose C_20_-terminal glucosyl residue than C_3_/C_6_-terminal. Conversely, C_3_-terminal glucosyl residue of OA-type ginsenosides was easier to occur cleavage reactions than C_28_-terminal glucosyl residue. M[oreover](javascript:;), [M+NH_4_]^+^ and [M+H]^+^ ion of ginsenosides was easier to occur in-source fragmentation and generated low-mass product ion clusters in which the protonated sapogenins eliminate H_2_O successively. Next, spectrum of five main type ginsenosides (PPD, PPT, OA, OT and acyl-substituted type) was elucidated through a typical representative compound respectively.

Ginsenoside Rb_1_ (M234) is a typical representative of PPD-type ginsenosides, which gave [M-H+FA]^-^, [M+Na]^+^ and [M+NH_4_]^+^ ions at m/z 1153, m/z 1131 and m/z 1226, respectively. In the negative ion mode, the FA-adduct precursor ion (m/z 1153) fragmented into m/z 1107 ([M-H]^–^), m/z 945 ([M-H-Glc]^–^), m/z 783 ([M-H-2Glc]^–^), m/z 621 ([M-H-3Glc]^–^) and m/z 459 ([M-H-4Glc]^–^; the sapogenin ion of PPD). The m/z 459 ([PPD-H]^–^) further fragmented into m/z 375 ([PPD‒H‒C_6_H_12_]^‒^; a typical secondary product ion of deprotonated PPD by cleavage of C_20_-C_22_ chemical bond). And the product ions at m/z 221 could be diagnostic for the presence of GlcGlc. In the positive ion mode, odium-adduct precursor ion (m/z 1131) yielded abundant [M+Na-GlcGlc]^+^ product ion at m/z 789 by losing 20-GlcGlc (342 DA) and abundant [GlcGlc+Na]^+^ product ion at m/z 365 by losing both 20-GlcGlc and 3-GlcGlc. Moreover, [M+NH_4_]^+^ generated product ions at m/z 407/425/443 which were the secondary fragments of the protonated PPD sapogenin ([PPD+H]^+^) by consecutive neutral eliminations of H_2_O. Thus, Compound 234 was preliminarily characterized as PPD-20-Glc-Glc-3-Glc-Glc, which further was identified by reference standards of Ginsenoside Rb_1_.


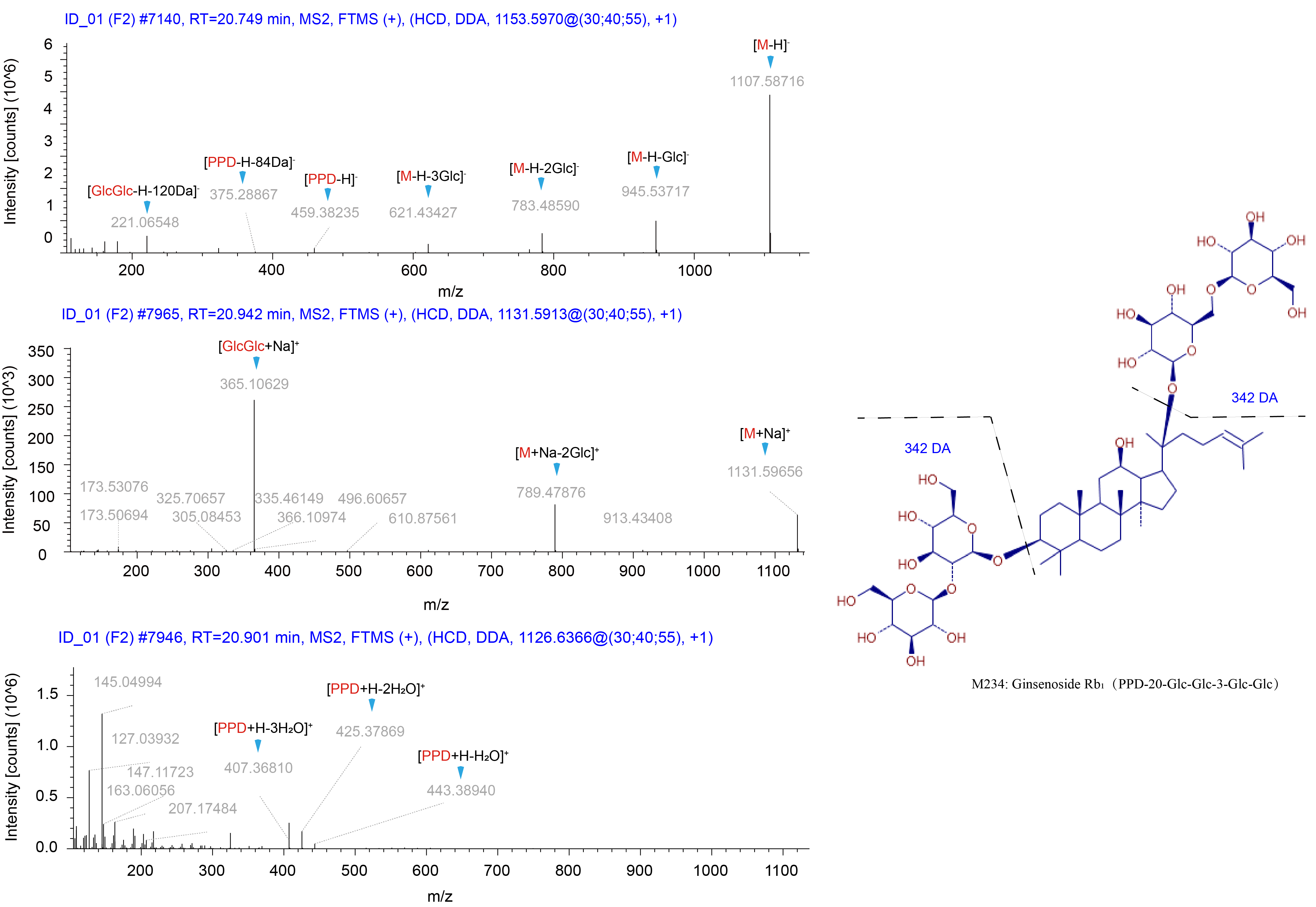


FIGURE S2 The MS/MS spectra of Ginsenoside Rb_1_ (M234)

Ginsenoside Re (M159) is a typical representative of PPT-type ginsenosides, which gave [M-H+FA]^-^, [M+Na]^+^ and [M+NH_4_]^+^ ions at m/z 991, m/z 969 and m/z 964, respectively. In the negative ion mode, the FA-adduct precursor ion (m/z 991) fragmented into m/z 945 ([M-H]^–^), m/z 783 ([M-H-Glc]^–^), m/z 621 ([M-H-Glc-Rha]^–^) and m/z 475 ([M-H-2Glc-Rha]^–^; the sapogenin ion of PPT). The m/z 475 ([PPT-H]^–^) further fragmented into m/z 391 ([PPD‒H‒C_6_H_12_]^‒^; a typical secondary product ion of deprotonated PPT by cleavage of C_20_-C_22_ chemical bond). In the positive ion mode, odium-adduct precursor ion (m/z 969) yielded abundant [M+Na-Glc]^+^ product ion at m/z 789 by losing 20-Glc (162 DA) and [GlcRha+Na]^+^ product ion at m/z 349 by losing 6-GlcRha. Moreover, [M+NH_4_]^+^ generated product ions at m/z 405/423/441 which were the secondary fragments of the protonated PPT sapogenin ([PPT+H]^+^) by consecutive neutral eliminations of H_2_O. Thus, Compound 159 was preliminarily characterized as PPT-20-Glc-6-Glc-Rha, which further was identified by reference standards of Ginsenoside Re.


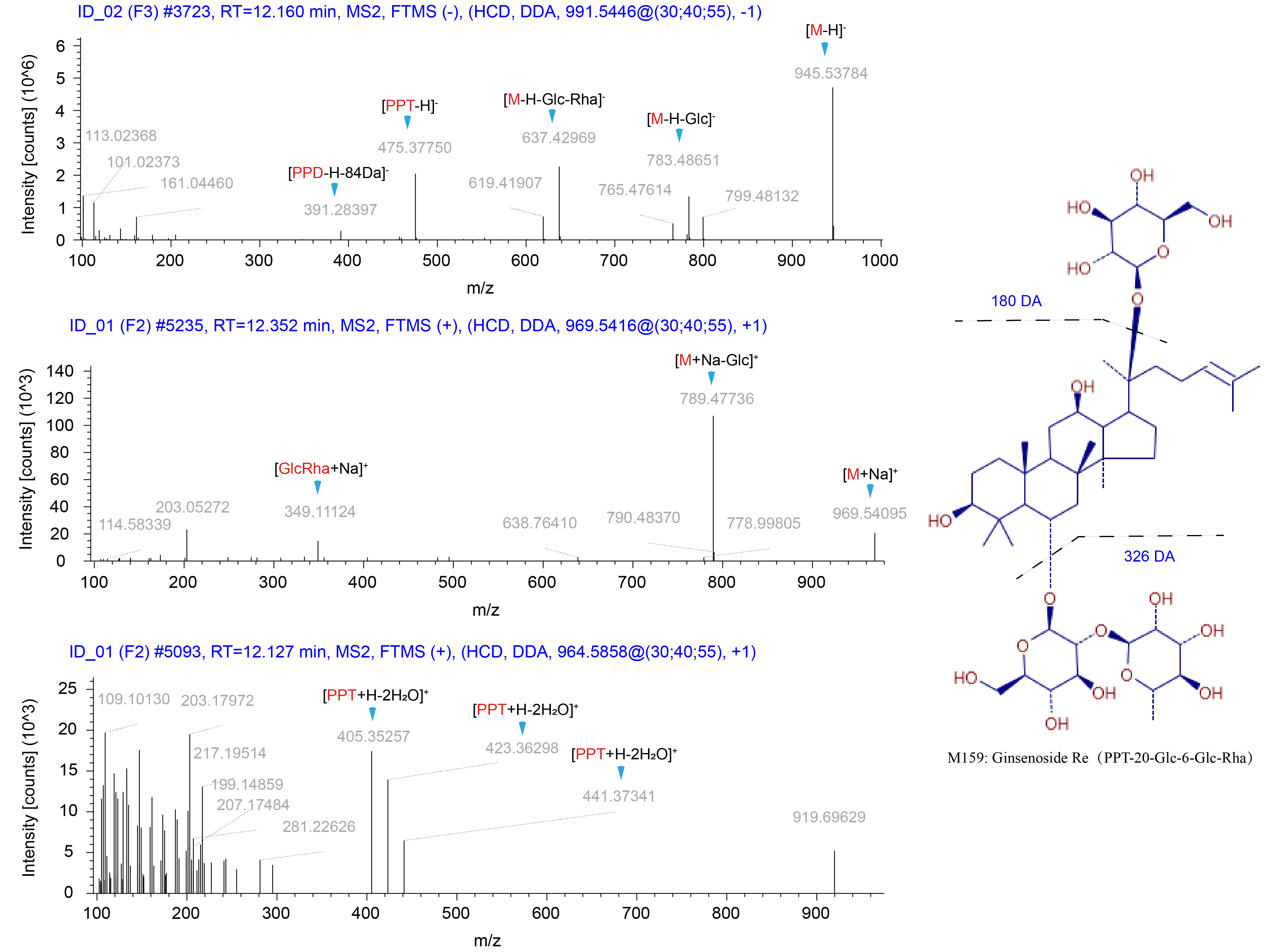


FIGURE S3 The MS/MS spectra of Ginsenoside Re (M159)

Pseudo-Ginsenoside F_11_ (M206) is a typical representative of OT-type ginsenosides, which gave [M-H+FA]^-^, [M+Na]^+^ and [M+H]^+^ ions at m/z 845, 823 and 801, respectively. In the negative ion mode, the FA-adduct precursor ion (m/z 845) fragmented into m/z 799 ([M-H]^–^), m/z 653 ([M-H-Rha]^–^) and m/z 491 ([M-H-Glc-Rha]^–^); the sapogenin ion of OT). And the product ions at m/z 205 could be diagnostic for the presence of GlcRha. In the positive ion mode, odium-adduct precursor ion (m/z 823) yielded [M+Na-Glc-Rha]^+^ product ion at m/z 497 by losing 6-Glc-Rha (326 DA). Moreover, [M+H]^+^ generated product ions at m/z 421/439/457 which were the secondary fragments of the protonated OT sapogenin ([OT+H]^+^) by consecutive neutral eliminations of H_2_O. Thus, Compound 206 was preliminarily characterized as OT-6-Glc-Rha, which further was identified by reference standards of Pseudo-Ginsenoside F_11_.


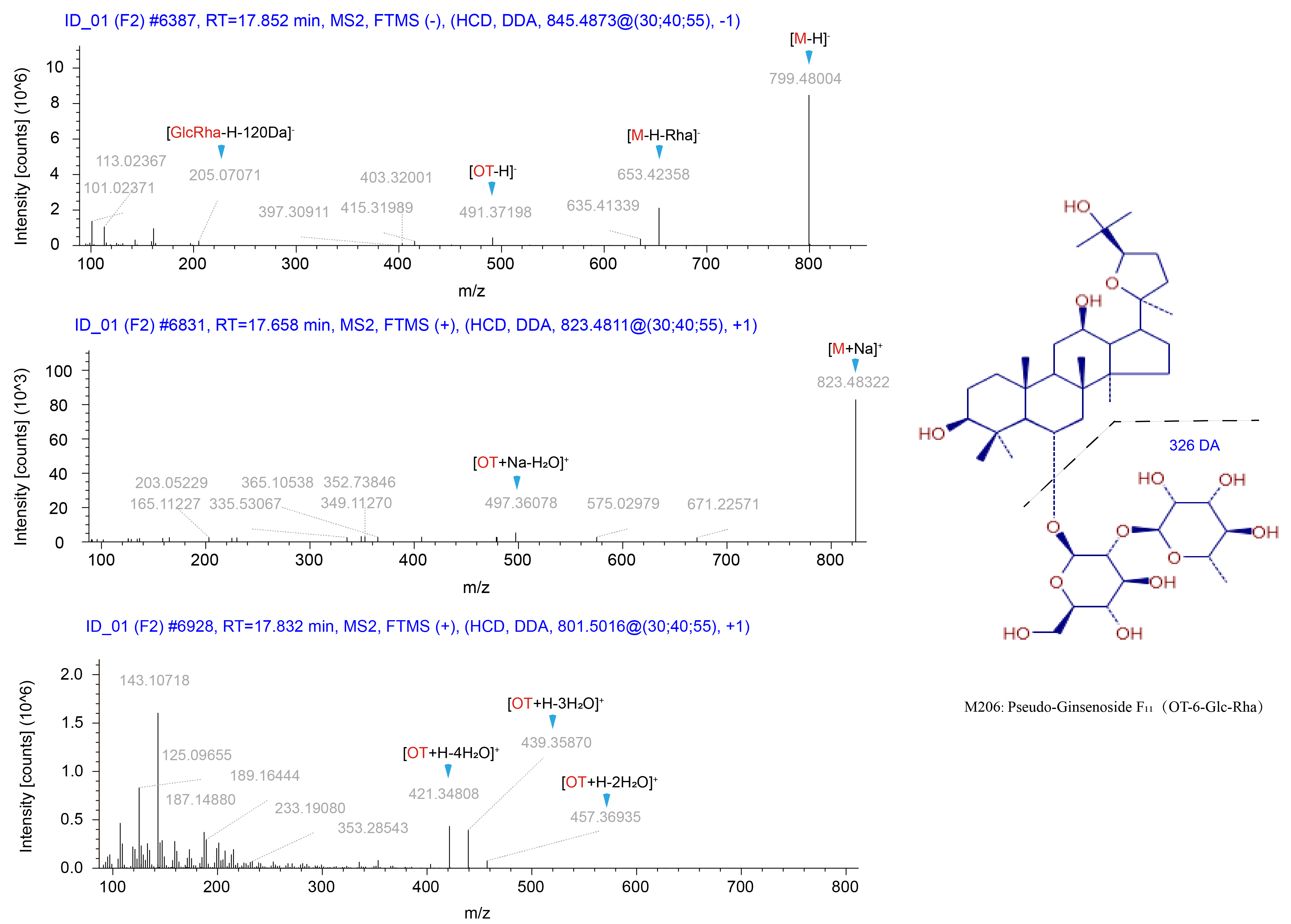


FIGURE S4 The MS/MS spectra of Pseudo-Ginsenoside F_11_ (M206)

Ginsenoside Ro (M246) is a typical representative of OA-type ginsenosides, which gave [M-H]^-^, [M+Na]^+^ and [M+NH_4_]^+^ ions at m/z 955, 979 and 974, respectively. In the negative ion mode, the precursor ion (m/z 955) fragmented into m/z 793 ([M-H-Glc]^–^), m/z 613 ([M-H-2Glc-H_2_O]^–^), m/z 569 ([M-H-2Glc-CO_2_-H_2_O]^–^) and m/z 455 ([M-H-GluA-2Glc]^–^; the sapogenin ion of OA). In the positive ion mode, odium-adduct precursor ion (m/z 979) yielded abundant [M+Na-GluA-Glc]^+^ product ion at m/z 641 by losing 3-GluA-Glc (338 DA). Meanwhile, 3-GluA-Glc could be observed at m/z 361 ([GluAGlc+Na]^+^). Moreover, [M+NH_4_]^+^ generated product ions at m/z 393/439 which were the secondary fragments of the protonated OA sapogenin ([OA+H]^+^) by consecutive neutral eliminations of H_2_O and HCOOH. Thus, Compound 246 was primarily characterized as OA-3-GluA-Glc-28-Glc, which further was identified by reference standards of Ginsenoside Ro.


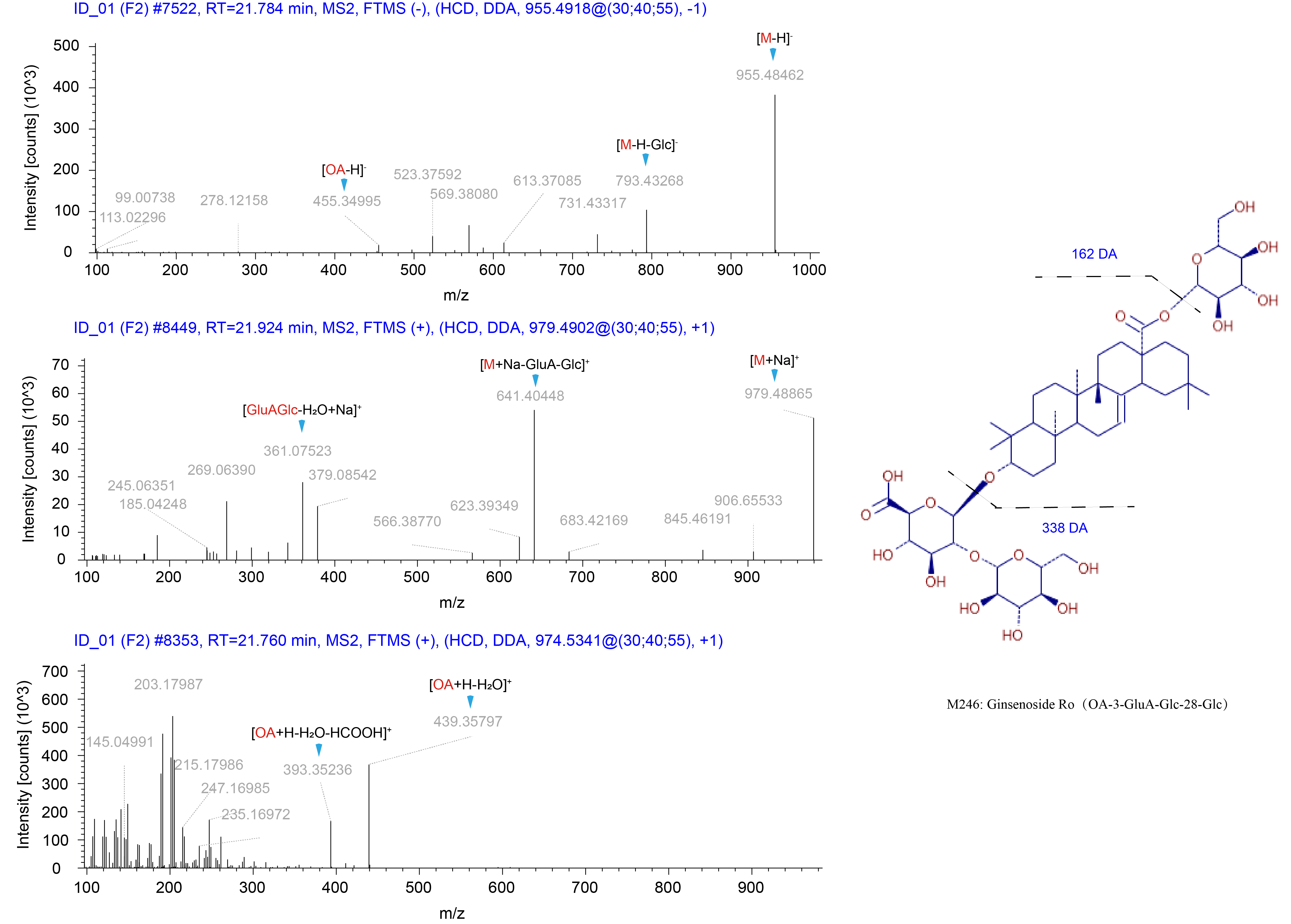


FIGURE S5 The MS/MS spectra of Ginsenoside Ro (M246)

Compound 241 (t_R_=21.34 min) was selected as a representative to illustrate fragmentation behaviors of malonylated ginsenosides, which gave [M-H]^-^,[M+Na]^+^ and [M+NH_4_]^-^ ions at m/z 1193, 1217 and 1212, respectively. In the negative ion mode, the precursor ion (m/z 1193) fragmented into m/z 1149 ([M-H-CO_2_]^–^), m/z 1107 ([M-H-Mal]^–^), m/z 945 ([M-H-Glc-Mal]^–^), m/z 783 ([M-H-2Glc-Mal]^–^), m/z 621 ([M-H-3Glc-Mal]^–^) and m/z 459 ([M-H-4Glc-Mal]^–^; the sapogenin ion of PPD). And the product ions at m/z 221 could be diagnostic for the presence of GlcGlc. In the positive ion mode, odium-adduct precursor ion (m/z 1217) yielded abundant [M+Na-GlcGlc]^+^ product ion at m/z 875 by losing 20-GlcGlc (342 DA) and [GlcGlc+Na]^+^ product ion at m/z 451 by losing 3-GlcGlcMal. Moreover, [M+NH_4_]^+^ generated product ions at m/z 407/425/443 which were the secondary fragments of the protonated PPD sapogenin ([PPD+H]^+^) by consecutive neutral eliminations of H_2_O. Thus, Compound 241 was primarily characterized as PPD-20-Glc-Glc-3-Glc-Glc-Mal.


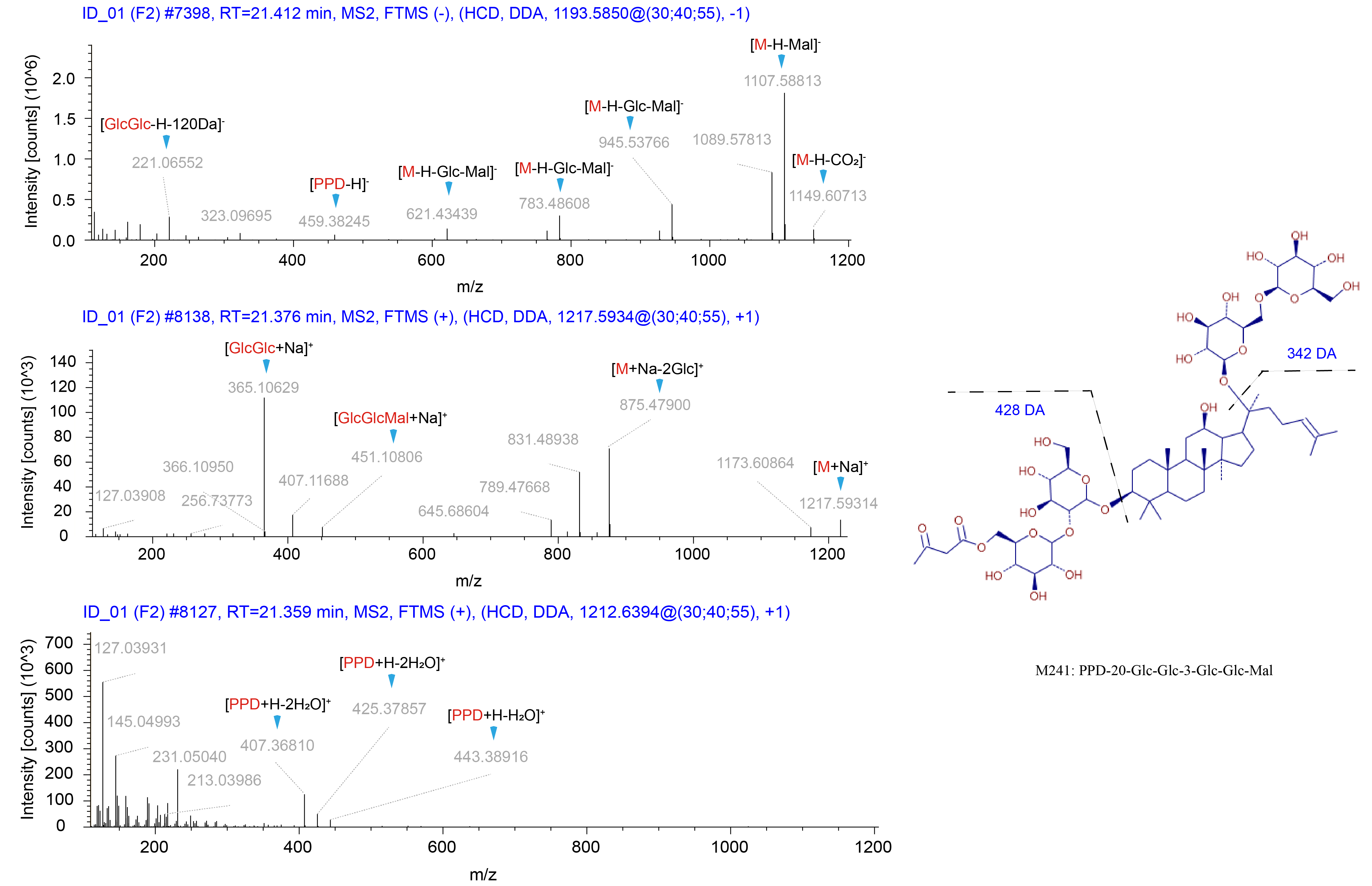


FIGURE S6 The MS/MS spectra of Compound 241

**3. Identification of other metabolites**

Other metabolites were recognized by 18 standards and mzCloud with match scores greater than 70. Here, Arginine (M3), Citric acid (M42), Azelaic acid (M148), Maltose (M25), Chlorogenic acid (M92) and Guanosine monophosphate (M39) is selected as a representative of amino acids and derivatives, organic acids and derivatives, lipids, carbohydrates and derivatives, phenols, nucleotides and derivatives respectively. The comparison MS/MS spectra between sample and mzCloud database was shown in FIGURE S7.


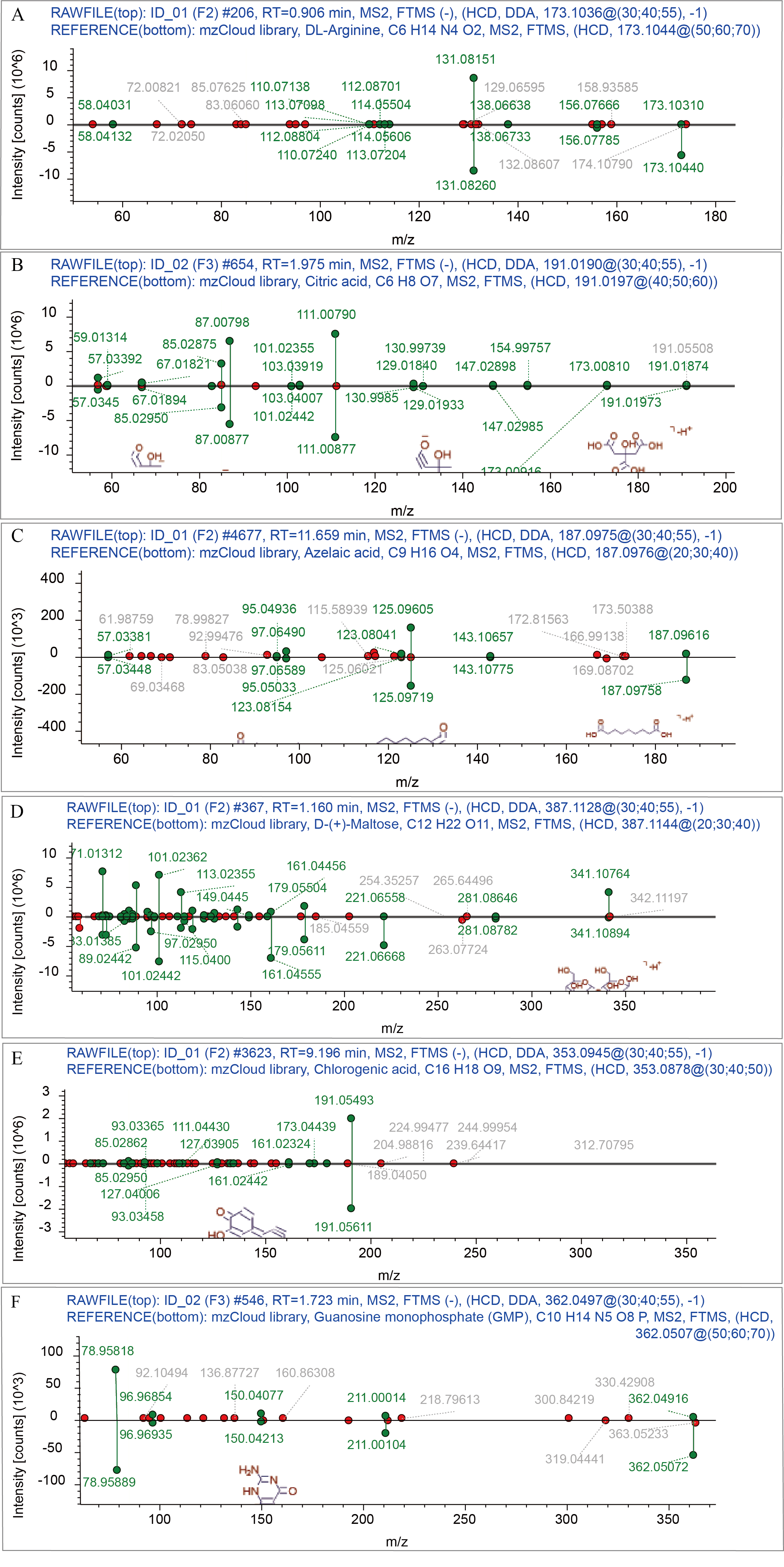


FIGURE S7 MS/MS spectrums of Arginine (A), Citric acid (B), Azelaic acid (C), Maltose (D), Chlorogenic acid (E) and Guanosine monophosphate (F) in sample (upper panel) and mzCloud (lower panel)


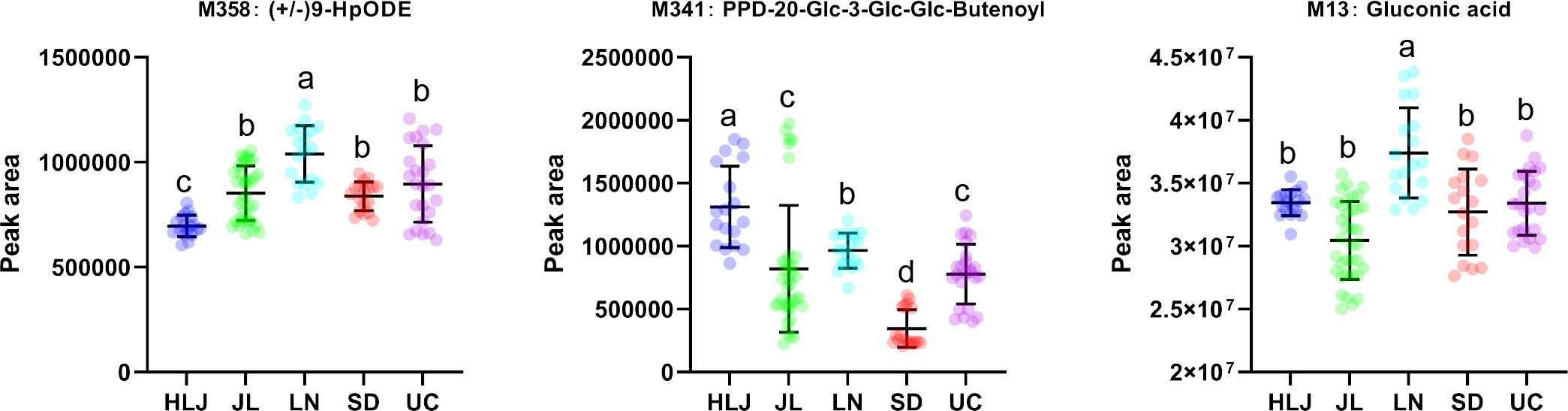


FIGURE S8 Relative content of compounds with the top 3 score VIP from OPLS-DA model of five producing areas

Shi, X. J., Yang, W. Z., Qiu, S., Yao, C. L., Shen, Y., Pan, H. Q., Bi, Q. R., Yang, M., Wu, W. Y., & Guo, D. A. (2017). An in-source multiple collision-neutral loss filtering based nontargeted metabolomics approach for the comprehensive analysis of malonyl-ginsenosides from *Panax ginseng*, *P*. *quinquefolius*, and *P*. *notoginseng*. *Analytica Chimica Acta*, *952*, 59-70. <https://doi.org/10.1016/j.aca.2016.11.032>

Wang, H., Zhang, C., Zuo, T., Li, W., Jia, L., Wang, X., Qian, Y., Guo, D., & Yang, W. (2019). In-depth profiling, characterization, and comparison of the ginsenosides among three different parts (the root, stem leaf, and flower bud) of *Panax quinquefolius* L. by ultra-high performance liquid chromatography/quadrupole-Orbitrap mass spectrometry. *Analytical and Bioanalytical Chemistry*, *411*, 7817-7829. https://doi.org/10.1007/s00216-019-02180-8

Yang, W. Z., Shi, X. J., Yao, C. L., Huang, Y., Hou, J. J., Han, S. M., Feng, Z. J., Wei, W. L., Wu, W. Y., & Guo, D. A. (2020). A novel neutral loss/product ion scan-incorporated integral approach for the untargeted characterization and comparison of the carboxyl-free ginsenosides from *Panax ginseng*, *Panax quinquefolius*, and *Panax notoginseng*. *Journal of Pharmaceutical and Biomedical Analysis*, *177*, 112813. https://doi.org/10.1016/j.jpba.2019.112813

Yang, W. Z., Ye, M., Qiao, X., Liu, C. F., Miao, W. J., Bo, T., Tao, H. Y., & Guo, D. A. (2012). A strategy for efficient discovery of new natural compounds by integrating orthogonal column chromatography and liquid chromatography/mass spectrometry analysis: Its application in *Panax ginseng*, *Panax quinquefolium* and *Panax notoginseng* to characterize 437 potential new ginsenosides. *Analytica Chimica Acta*, *739*, 56-66. https://doi.org/10.1016/j.aca.2012.06.017

Zhang, C. X., Wang, X. Y., Lin, Z. Z., Wang, H. D., Qian, Y. X., Li, W. W., Yang, W. Z., & Guo, D. A. (2020). Highly selective monitoring of in-source fragmentation sapogenin product ions in positive mode enabling group-target ginsenosides profiling and simultaneous identification of seven Panax herbal medicines. *Journal of Chromatography A*, *1618*, 460850. https://doi.org/10.1016/j.chroma.2020.460850

Zuo, T., Zhang, C., Li, W., Wang, H., Hu, Y., Yang, W., Jia, L., Wang, X., Gao, X., & Guo, D. (2020). Offline two-dimensional liquid chromatography coupled with ion mobility-quadrupole time-of-flight mass spectrometry enabling four-dimensional separation and characterization of the multicomponents from white ginseng and red ginseng. *Journal of Pharmaceutical Analysis*, *10*, 597-609. https://doi.org/10.1016/j.jpha.2019.11.001
